# Insulin resistance and white adipose tissue inflammation are uncoupled in energetically challenged Fsp27-deficient mice

**DOI:** 10.1038/ncomms6949

**Published:** 2015-01-07

**Authors:** Linkang Zhou, Shi-Young Park, Li Xu, Xiayu Xia, Jing Ye, Lu Su, Kyeong-Hoon Jeong, Jang Ho Hur, Hyunhee Oh, Yoshikazu Tamori, Cristina M. Zingaretti, Saverio Cinti, Jesús Argente, Miao Yu, Lizhen Wu, Shenghong Ju, Feifei Guan, Hongyuan Yang, Cheol Soo Choi, David B. Savage, Peng Li

**Affiliations:** 1 MOE Key Laboratory of Bioinformatics and Tsinghua-Peking Center for Life Sciences, School of Life Sciences, Tsinghua University, Beijing 100084, China; 2 Korea Mouse Metabolic Phenotyping Center, Lee Gil Ya Cancer and Diabetes Institute, Gachon University, Incheon 406-840, Korea; 3 Key Laboratory for Feed Biotechnology of the Ministry of Agriculture, Feed Research Institute, Chinese Academy of Agricultural Sciences, Beijing 100081, China; 4 Department of Pathology, The Fourth Military Medical University, Xi’an 710032, China; 5 Division of Diabetes and Endocrinology, Department of Internal Medicine, Kobe University Graduate School of Medicine, Kobe 650-0017, Japan; 6 Division of Metabolism and Endocrinology, Department of Internal Medicine, Chibune General Hospital, Osaka 555-0001, Japan; 7 Department of Experimental and Clinical Medicine-Obesity Center, United Hospitals-University of Ancona, Ancona 60020, Italy; 8 Department of Pediatrics and Pediatric Endocrinology, Hospital Infantil Universitario Niño Jesús, Madrid E-28009, Spain; 9 Instituto de Investigación La Princesa, Madrid E-28009, Spain; 10 Department of Pediatrics, Universidad Autónoma de Madrid, Madrid E-28009, Spain; 11 CIBER Fisiopatología de la obesidad y nutrición, Instituto de Salud Carlos III, Madrid E-28009, Spain; 12 Jiangsu Key Laboratory of Molecular and Functional Imaging, Department of Radiology, Zhongda Hospital, Southeast University, Nanjing 210009, China; 13 Key Laboratory of Human Disease Comparative Medicine, Ministry of Health, Institute of Laboratory Animal Science, Chinese Academy of Medical Sciences and Comparative Medical Center, Peking Union Medical College, Beijing 100084, China; 14 School of Biotechnology and Biomolecular Sciences, University of New South Wales, Sydney, New South Wales 2052, Australia; 15 Department of Internal Medicine, Gachon University Gil Medical Center, Incheon 405-760, Korea; 16 University of Cambridge Metabolic Research Laboratories, Wellcome Trust-Medical Research Council Institute of Metabolic Science, Cambridge CB2 0QQ, UK

## Abstract

Fsp27 is a lipid droplet-associated protein almost exclusively expressed in adipocytes where it facilitates unilocular lipid droplet formation. In mice, *Fsp27* deficiency is associated with increased basal lipolysis, ‘browning’ of white fat and a healthy metabolic profile, whereas a patient with congenital CIDEC deficiency manifested an adverse lipodystrophic phenotype. Here we reconcile these data by showing that exposing *Fsp27*-null mice to a substantial energetic stress by crossing them with *ob/ob* mice or BATless mice, or feeding them a high-fat diet, results in hepatic steatosis and insulin resistance. We also observe a striking reduction in adipose inflammation and increase in adiponectin levels in all three models. This appears to reflect reduced activation of the inflammasome and less adipocyte death. These findings highlight the importance of Fsp27 in facilitating optimal energy storage in adipocytes and represent a rare example where adipose inflammation and hepatic insulin resistance are disassociated.

## Abstract (web summary)

Fsp27 mediates ‘fusion’ of lipid droplets in mouse adipose tissue. Here, the authors investigate the physiological consequences of loss of Fsp27 in three different mouse models of ‘energetic overload’, and observe hepatic steatosis and insulin resistance but reduced adipose tissue inflammation.

## Graphical abstract

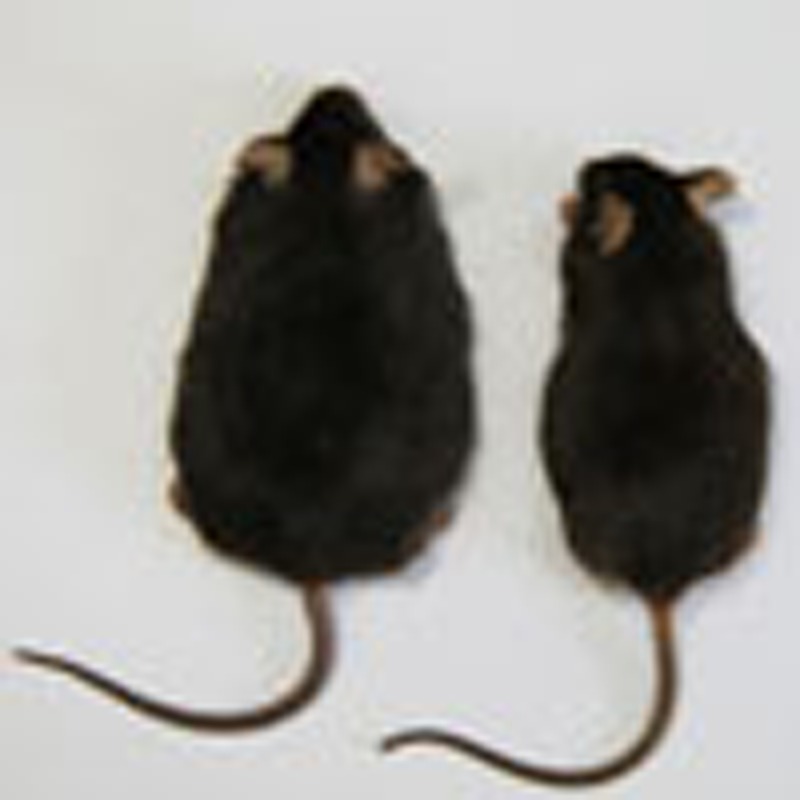

CIDE proteins including Cidea, Cideb and Fsp27 (Cidec in humans), have emerged as key regulators of lipid droplet (LD) morphology and function in adipocytes and hepatocytes^1^,^2^,^3^. Cidea is predominantly expressed in brown adipocytes, Cideb in hepatocytes and Fsp27 almost exclusively in white adipocytes in healthy wild-type (WT) mice^3^. In humans, one potentially important difference is that Cidea is also expressed in white adipocytes^2^,^4^. Fsp27 and Cidea are also expressed in steatotic livers^5^,^6^. Both Fsp27 and Cidea localize on the surface of LDs^7^,^8^,^9^, are particularly enriched at LD contact sites and appears to promote a unique form of ‘LD fusion’^9^,^10^. Perilipin1 enhances Fsp27-mediated LD fusion in white adipocytes^11^. Fsp27 knockdown studies in cultured 3T3L1 adipocytes^8^ and *in vivo* evidence from two independently generated *Fsp27*-null lines^12^,^13^ and from a single human patient with a homozygous loss-of-function premature stop mutation^14^ clearly supports the notion that Fsp27 is required for the formation of a large unilocular LD in white adipocytes. The presence of multilocular LDs in *Fsp27*-deficient adipocytes is in turn consistently associated with increased lipolysis^8^,^13^, presumably as a result of a considerable increase in the LD surface area accessible to lipases.

White adipocytes are uniquely adapted to store surplus energy in unilocular LDs and to quantitatively release non-esterified fatty acids for oxidation by other metabolically active tissues. Excessive lipid storage in white adipose tissue (WAT) results in the development of obesity and ultimately its related complications including insulin resistance, non-alcoholic fatty liver disease (NAFLD) and cardiovascular disease^15^,^16^,^17^. Interestingly, lipodystrophic states, which are characterized by reduced fat mass and defective lipid storage in adipose tissue, are strongly associated with ectopic fat deposition and an almost identical constellation of metabolic problems to those associated with obesity. In humans, at least 12 different genetic subtypes of lipodystrophy have been reported, and these almost invariably result in NAFLD, dyslipidemia and insulin resistance, which frequently leads to diabetes^18^,^19^,^20^. In contrast to this human paradigm, several mouse models including some specifically designed to mimic human lipodystrophies^21^, as well as others independently generated in direct attempts to create lipodystrophic mouse models or simply to understand the *in vivo* consequences of targeted genetic perturbations, have appeared to display ‘lean and healthy’ metabolic phenotypes without fatty liver disease and insulin resistance^19^,^22^.

Although the importance of Fsp27 in mediating the formation of a unilocular LD in adipocytes is very clear and we have suggested a plausible mechanistic basis for this function^9^,^11^, the physiological importance of this activity is much less certain. In mice and the human patient, *Fsp27* deficiency resulted in a significant reduction in total fat mass, but the systemic consequences of this adipose phenotype were very different. Whereas the human patient manifested a typical ‘lipodystrophic’ phenotype characterized by ectopic lipid accumulation in the liver, that is, NAFLD, dyslipidemia and insulin-resistant diabetes^14^, both the knockout mouse models appeared to be protected against insulin resistance^12^,^13^.

In the current studies, we endeavour to clarify the physiological importance of Fsp27, particularly in relation to its role in optimizing lipid storage and insulin sensitivity. Whereas mice are typically housed at temperatures below thermoneutrality and fed diets with <10% fat content, humans tend to ensure that their environs are thermoneutral and habitually consume diets with a far greater fat content. These differences are expected to result in significant differences in the need to store surplus fat, particularly in mouse models where adipose tissue insulation is reduced. Thus, to subject the *Fsp27*-deficient mice to a greater energetic burden, the mice are crossed with *leptin*-deficient *ob/ob* mice or challenged with prolonged high-fat feeding. As brown adipose tissue (BAT) could conceivably aid adaptation to any deficiency in WAT lipid storage, especially in mice with less fat mass housed in a relatively cold environment where thermogenesis is typically enhanced, we also seek to ascertain the importance of thermogenic BAT in these mice by crossing the Fsp27 knockouts with BATless mice^23^.

## Results

### Reduced fat mass and inflammation in ob/ob/Fsp27^−/−^ mice

**Figure 1:**
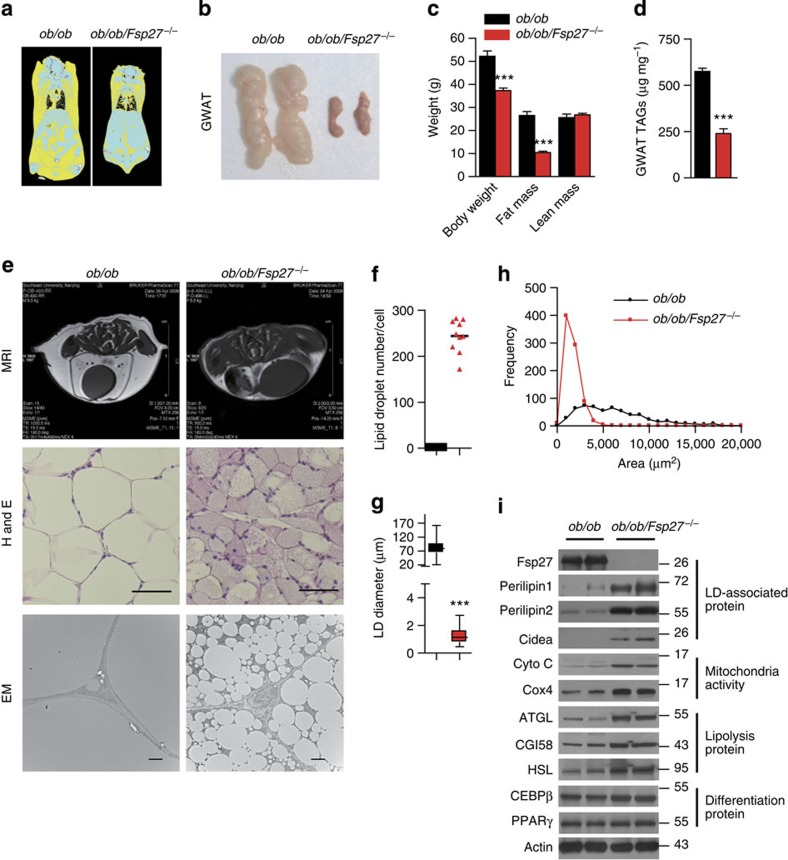
Reduced fat mass in the *ob/ob/Fsp27*^−/−^ mice. Four-month-old *ob/ob* and *ob/ob/Fsp27*^−/−^ mice were maintained on a chow diet for the analyses shown in (**a**–**i**). (**a**) computed tomography (CT) scan analysis of the mice; fat is shown in yellow. (**b**) GWAT of *ob/ob* and *ob/ob/Fsp27*^−/−^ mice. (**c**) Body composition of *ob/ob* (*n*=5) and *ob/ob/Fsp27*^−/−^ mice (*n*=6). (**d**) Decreased TAG content in the GWAT of the *ob/ob* and *ob/ob/Fsp27*^−/−^ mice (*n*=5). (**e**) Abdominal magnetic resonance imaging (MRI) of *ob/ob* and *ob/ob/Fsp27*^−/−^ mice (upper panel). Fat is shown in white in these MRI images. GWAT morphology (middle and lower panels). H&E, haematoxylin and eosin staining; EM, electron microscope. Scale bar, 64 and 2 μm for H&E staining and EM, respectively. (**f**) LD number per adipocyte in *ob/ob* and *ob/ob/Fsp27*^−/−^ mice. The number of LDs in ten adipocytes was measured. (**g**) The average LD diameter in the GWAT of *ob/ob* and *ob/ob/Fsp27*^−/−^ mice. The diameter of LDs in ten adipocytes was measured. (**h**) The distribution of fat cell size in the GWAT of *ob/ob* and *ob/ob/Fsp27*^−/−^ mice. The fat cell area from 400 adipocytes was measured. (**i**) A representative western blotting showing the expression pattern of Fsp27, Perilipin1, Perilipin2, Cidea, Cyto C, Cox4, ATGL, CGI58, HSL, CEBPβ and PPARγ in the GWAT of *ob/ob* and *ob/ob/Fsp27*^−/−^ mice. Actin was used as a loading control. Quantitative data are presented as mean±s.e.m. Significance was established using a two-tailed Student’s *t*-test. Differences were considered significant at *P*<0.05. ***P<0.001.

*Fsp27*-null mice were crossed with *leptin*-deficient (*ob/ob*) mice to generate doubly-deficient mice (*ob/ob/Fsp27*^−/−^). All the offspring were viable, born at the expected frequencies and of similar length to their *ob/ob* littermates (Supplementary Fig. 1a–c). *ob/ob/Fsp27*^−/−^ mice weighed ~30% less than *ob/ob* mice (Table 1 and Supplementary Fig. 1a), with the bulk of this difference being due to substantial reductions in the volume of both subcutaneous and visceral fat (Fig. 1a–d and Table 1). Lean mass was similar in both groups (Fig. 1c). The major determinant of the differences in body weight appears to be increased energy expenditure in the *ob/ob/Fsp27*^−/−^ mice (Supplementary Fig. 1d), as food intake tended to be greater in the *ob/ob/Fsp27*^−/−^ mice (Supplementary Fig. 1e). Consistent with the role of Fsp27 in regulating LD fusion, white adipocytes of *ob/ob/Fsp27*^−/−^ mice were smaller and contained small multilocular LDs (Fig. 1e–h). Expression levels of several LD-associated proteins (Perilipin1, Perilipin2 and Cidea) were significantly increased (Fig. 1i), presumably due to the relative increase in LD-associated surface area. Proteins involved in mitochondrial oxidative phosphorylation, fatty acid oxidation (Cyto C and Cox4) and lipolysis (ATGL, CGI58 and HSL) were all increased, whereas levels of key adipogenic transcription factors (CEBPβ and PPARγ) were similar in *ob/ob/Fsp27*^−/−^and *ob/ob* mice (Fig. 1i). Plasma levels of glycerol were increased in *ob/ob/Fsp27*^−/−^ mice, reflecting increased lipolysis as previously observed in *Fsp27*-deficient mice^12^,^13^ (Table 1).

**Figure 2:**
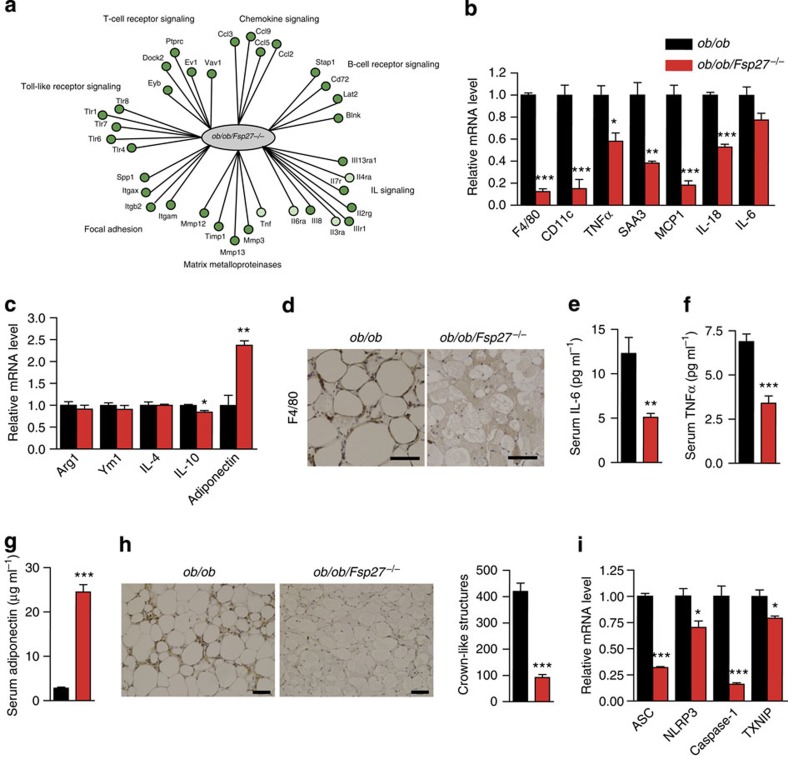
Reduced WAT inflammatory response in *ob/ob/Fsp27*^−/−^ mice. Four-month-old chow-fed *ob/ob* and *ob/ob/Fsp27*^−/−^ mice were used for the analyses in (**a**–**i**). (**a**) Gene expression profiling in the GWAT of *ob/ob* and *ob/ob/Fsp27*^−/−^ mice. Green circles represent downregulated genes in the *ob/ob/Fsp27*^−/−^ mice compared with *ob/ob* mice. The intensity of the green colour indicates the extent of downregulation. (**b**) Relative mRNA expression of *F4/80*, *CD11c* and other pro-inflammatory genes, or (**c**) anti-inflammatory genes in the GWAT of *ob/ob* and *ob/ob/Fsp27*^−/−^ mice (*n*=4 per group). (**d**) F4/80 immunohistochemical analysis in the GWAT of *ob/ob* and *ob/ob/Fsp27*^−/−^ mice. Scale bar, 64 μm. (**e**) Serum concentration of IL-6 (*n*=7 per group). (**f**) Serum concentration of TNFα (*n*=7 for *ob/ob* and *n*=9 for *ob/ob/Fsp27*^−/−^). (**g**) Serum concentration of adiponectin (*n*=8 per group). (**h**) TNFα immunohistochemical analysis in the GWAT of *ob/ob* and *ob/ob/Fsp27*^−/−^ mice showing the CLSs (left). Right: statistic analysis of the CLSs per 1,000 adipocytes. Scale bar, 50 μm. (**i**) Relative mRNA levels of *ASC*, *NLRP3*, *Caspase-1* and *TXNIP* in the GWAT of *ob/ob* and *ob/ob/Fsp27*^−/−^ mice (*n*=3 per group). Quantitative data are presented as mean±s.e.m. Significance was established using a two-tailed Student’s *t*-test. Differences were considered significant at *P*<0.05. *P<0.05, **P<0.01, ***P<0.001.

Next, we systematically analysed the gene expression profiles of gonadal WAT (GWAT) from *ob/ob/Fsp27*^−/−^ and *ob/ob* mice by microarray analysis, and observed that the expression of 8,000 genes were different in this depot. Wiki pathway analysis suggested that 23 of 162 Wiki pathways were significantly increased, whereas 39 pathways were significantly decreased in the GWAT of *ob/ob/Fsp27*^−/−^ mice compared with that in *ob/ob* mice (Supplementary Table 1). Importantly, expression levels of genes in the inflammatory response pathway, B- and T-cell receptor signalling pathway and chemokine signalling pathway were all markedly decreased (Fig. 2a and Supplementary Table 1). A similar comparison of gene expression data from GWAT of chow-fed *Fsp27*^−/−^ and WT mice revealed no significant differences in inflammatory pathways (Supplementary Table 2). Thus, the reduction in expression of genes involved in pro-inflammatory pathways was specific to the *ob/ob*/*Fsp27*^−/−^ mice.

We further validated these data by semi-quantitative reverse transcription–PCR and observed significantly lower expression for *F4/80* (a macrophage-specific marker), *Cd11c* (a marker of M1-like macrophages), *TNFα*, *SAA3*, *MCP1* and *IL-6* in the WAT of *ob/ob/Fsp27*^−/−^ mice (Fig. 2b). In contrast, expression levels of non-inflammatory M2 marker genes (*Arg1*, *Ym1* and *IL-4*) were similar between *ob/ob* and *ob/ob/Fsp27*^−/−^ mice (Fig. 2c). Immunohistochemical analyses also confirmed that F4/80 and tumour necrosis factor-α (TNFα) protein levels were significantly lower in the WAT of *ob/ob/Fsp27*^−/−^ mice (Fig. 2d,h). Consistent with reduced adipose tissue inflammation, plasma levels of interleukin (IL)-6 and TNFα were significantly reduced in *ob/ob/Fsp27*^−/−^ mice (Fig. 2e,f). In contrast, adiponectin messenger RNA expression in WAT and circulating adiponectin concentrations were considerably higher in the *ob/ob/Fsp27*^−/−^ mice (Fig. 2c,g).

Cinti *et al*.^24^,^25^ have previously shown that >90% of macrophages infiltrating WAT typically surround dead adipocytes forming crown-like structures (CLSs), and that the number of CLSs present in the WAT of *ob/ob* mice is significantly elevated. In *ob/ob/Fsp27*^−/−^mice, we observed a dramatic reduction in CLS compared with *ob/ob* mice (Fig. 2h). Exactly what causes cell death in hypertrophic adipocytes remains unclear, but Giordano *et al*.^26^ and others^27^ have suggested that NLRP3-dependent caspase-1 activation is likely to induce cell death by pyroptosis, a proinflammatory form of programmed cell death. In keeping with these data, we also observed significantly reduced expression of ASC, NLRP3, Caspase-1 and TXNIP in the adipose tissue of *ob/ob/Fsp27*^−/−^mice (Fig. 2i).

The reduction in WAT inflammation in *ob/obFsp27*^−/−^ mice prompted us to review the biopsy we had previously obtained from the patient with the E186X homozygous CIDEC mutation^14^. Careful review of this sample obtained from axilliary white fat indicated that this patient’s WAT was characterized by a mixed population of larger unilocular and smaller multilocular cells, and that the CLSs were only observed in relation to unilocular cells (Supplementary Fig. 2). These observations are consistent with the notion that the smaller multilocular *Fsp27*-null cells are less prone to macrophage recruitment and activation. Overall, these data strongly indicate that the WAT of *ob/obFsp27*^−/−^ mice is defective in storing lipid and manifests reduced chronic inflammation.

### Fatty liver and insulin resistance in ob/ob/Fsp27^−/−^ mice

**Figure 3:**
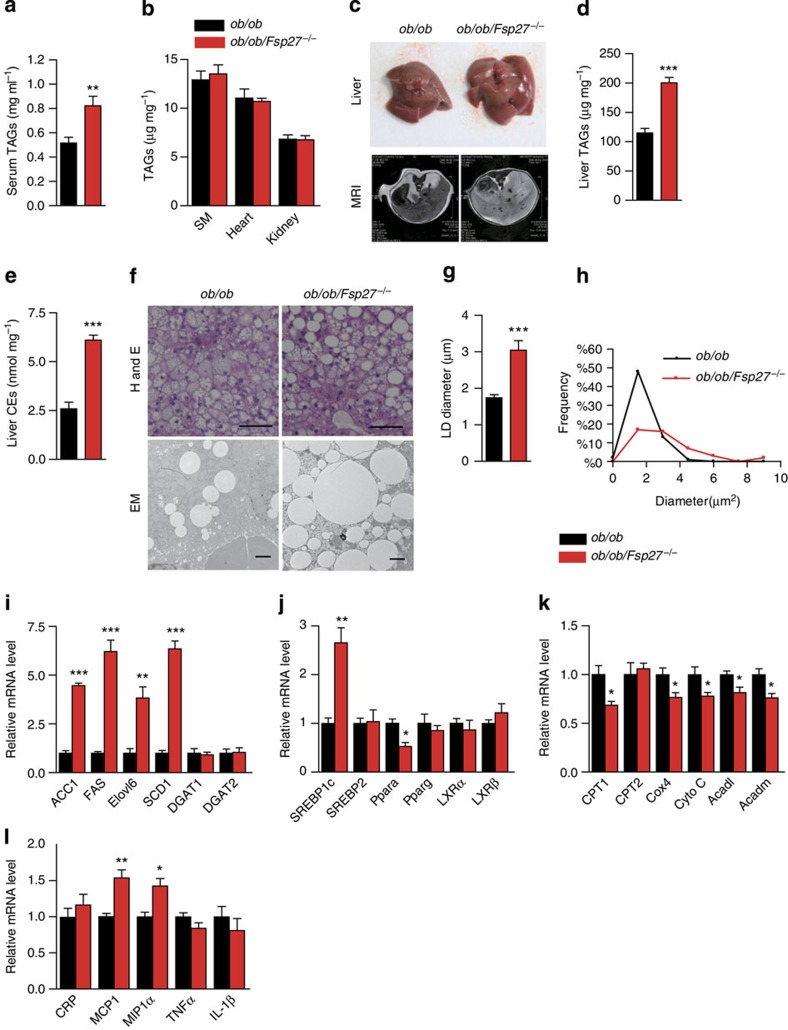
Hepatic steatosis in *ob/ob/Fsp27*^−/−^ mice. Four-month-old chow-fed *ob/ob* and *ob/ob/Fsp27*^−/−^ mice were used (**a**–**l**). (**a**) Serum TAG concentrations (*n*=6 per group). (**b**) TAG content in the skeletal muscle (SM, gastrocnemius), heart and kidney (*n*=5 per group). (**c**) Photograph of the liver (top panel) and magnetic resonance imaging analysis of the liver section (lower panel). (**d**) Liver TAG content (*n*=8 for *ob/ob* and *n*=10 for *ob/ob/Fsp27*^*−*/−^ mice). (**e**) Liver CE content (*n*=5 per group). (**f**) Liver histology of *ob/ob* and *ob/ob/Fsp27*^−/−^ mice. H&E, haematoxylin and eosin staining. EM, electron microscope image. Scale bar, 64 and 2 μm for HE and EM, respectively. (**g**) The average LD diameter in the liver of *ob/ob* and *ob/ob/Fsp27*^−/−^ mice. The diameter of LDs in 50 cells was measured. (**h**) The distribution of LD size in the liver of *ob/ob* and *ob/ob/Fsp27*^−/−^ mice. (**i**–**l**) Relative mRNA expression levels in the livers of *ob/ob* and *ob/ob/Fsp27*^−/−^ mice (*n*=4 per group). Quantitative data are presented as mean±s.e.m. Significance was established using a two-tailed Student’s *t*-test. Differences were considered significant at *P*<0.05.*P<0.05, **P<0.01, ***P<0.001.

As defective adipose lipid storage often results in increased circulating triglyceride (TAG) levels and ectopic lipid deposition, we measured TAG levels in the serum and in several other tissues. Serum TAG concentrations were found to be significantly higher in *ob/ob/Fsp27*^−/−^ mice compared with that in *ob*/*ob* mice (Fig. 3a). No difference in tissue weight and TAG levels were observed in the skeletal muscle (Gastrocnemius), heart or kidneys of *ob/ob* and *ob/ob/Fsp27*^−/−^ mice (Table 1 and Fig. 3b). However, the size and weight of livers of *ob/ob/Fsp27*^−/−^ mice were significantly greater than those of *ob/ob* mice (Fig. 3c and Table 1). Hepatic levels of TAG and cholesterol ester (CE) were significantly greater in *ob/ob/Fsp27*^−/−^ mice (Fig. 3d,e). Consistent with this, larger LDs were observed in the liver of *ob/ob/Fsp27*^−/−^ mice (Fig. 3f–h). The interscapular BAT depot was larger in the *ob/ob/Fsp27*^−/−^ mice than in the *ob/ob* mice (Table 1 and Supplementary Fig. 3a) and contained larger LDs and elevated levels of TAG (Supplementary Fig. 3b,c).

Expression levels of several genes involved in hepatic *de novo* lipogenesis (*ACC1*, *FAS*, *Elovl6* and *SCD1*), and their major transcriptional regulator SREBP1c, were found to be significantly increased in *ob/ob/Fsp27*^−/−^ mice (Fig. 3i,j). In contrast, expression levels of several genes involved in fatty acid oxidation and oxidative phosphorylation (*Ppara*, *CPT1*, *Cox4*, *Cyto C*, *ACADL* and *ACADM*) were all slightly decreased in the liver of *ob/ob/Fsp27*^−/−^mice (Fig. 3j,k). Expression levels of inflammatory genes such as *MCP1* and *MIP1α* were increased in the liver of *ob/ob/Fsp27*^−/−^ mice (Fig. 3l).

**Figure 4:**
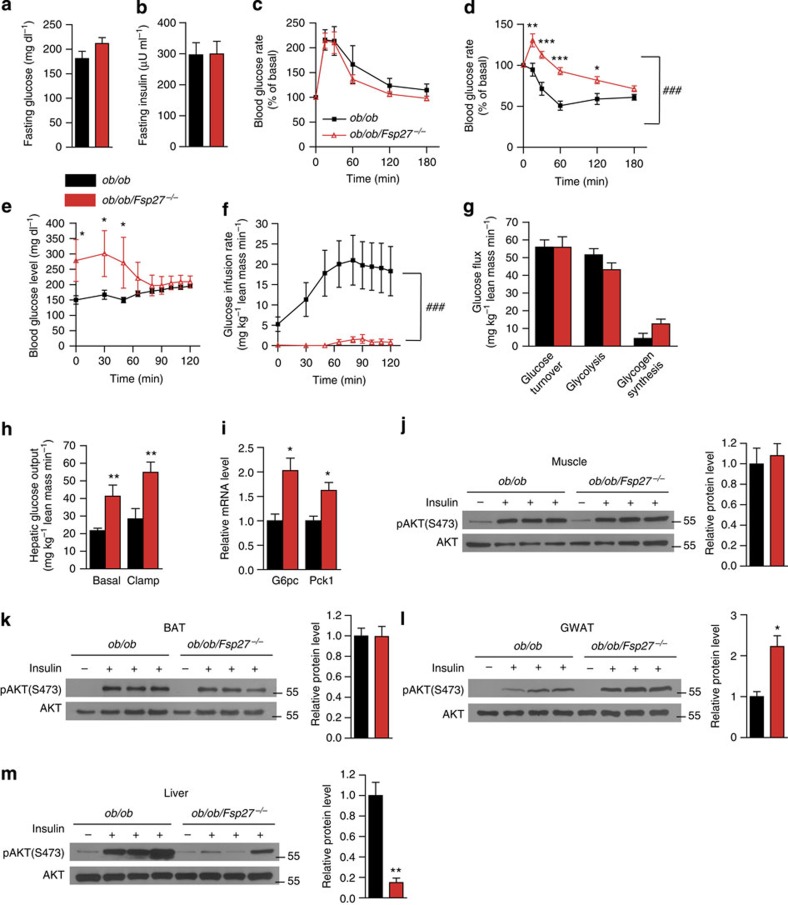
Insulin-resistant phenotype of *ob/ob/Fsp27*^−/−^ mice. (**a**) Fasting glucose and (**b**) fasting insulin concentrations of chow-fed 8.5-month-old *ob/ob* (*n*=6) and *ob/ob/Fsp27*^−/−^(*n*=7) mice. (**c**) Glucose tolerance tests (GTTs) and (**d**) ITTs in chow-fed 8.5-month-old *ob/ob* (*n*=7) and *ob/ob/Fsp27*^−/−^ (*n*=8) mice. Peripheral and hepatic insulin sensitivity were assessed in 4-month-old male mice using hyperinsulinaemic–euglycaemic clamps (**e**–**h**, *ob/ob, n*=11; *ob/ob/Fsp27*^−/−^, *n*=6). (**e**) Blood glucose concentrations during the clamp experiment. (**f**) Glucose infusion rates. (**g**) Peripheral glucose turnover. (**h**) Hepatic glucose output during basal and clamp conditions. (**i**) Relative mRNA level of *G6pc* and *Pck1* in the liver (*n*=4 per group). Insulin was injected in three pairs of anaesthetized 4-month-old male ob/ob and *ob/ob/Fsp27*^−/−^ mice. Representative images of basal and insulin-stimulated phospho-AKT (Ser473) levels in the muscle (**j**), BAT (**k**), gonadal fat (GWAT) (**l**) and liver (**m**). Quantitative data are presented as mean±s.e.m. Significance was established using a two-tailed Student’s *t*-test. Differences were considered significant at *P*<0.05.*P<0.05, **P<0.01, ***P<0.001. Two-way repeated-measurement analyses of variance were used to evaluate the data in Fig. 4c,d,f (^###^*P*<0.001 in this figure indicates that the two groups respond differently following the intervention).

*Fsp27*^−/−^ mice were previously shown to be glucose tolerant and more insulin sensitive than WT littermates when studied at 21–23 °C on a chow diet^12^,^13^. We have also previously reported that glucose tolerance was improved in *ob/ob/Fsp27*^−/−^ mice compared with *ob/ob* littermates when studied at a young age (10 weeks^12^). When studied at age 4 months, fasting glucose and insulin concentrations, glucose tolerance tests and insulin tolerance tests (ITTs) were again similar in *ob/ob/Fsp27*^−/−^ and *ob/ob* mice (Supplementary Fig. 3d–g). When studied at age 8.5 months, fasting glucose and insulin concentrations remained similar between *ob/ob/Fsp27*^−/−^ and *ob/ob* mice (Fig. 4a,b). Glucose tolerance was also similar in both groups (Fig. 4c). However, ITTs appeared to demonstrate insulin resistance (Fig. 4d) in the *ob/ob/Fsp27*^−/−^ group. To more precisely assess insulin sensitivity, we performed hyperinsulinaemic–euglycaemic clamps in these mice (at age 4 months). When infusing insulin at a constant rate (15 mU kg^−1^ min^−1^), the exogenous glucose infusion rate required to maintain euglycaemia was much lower in *ob/ob/Fsp27*^−/−^ mice compared with *ob*/*ob* mice, confirming reduced systemic insulin sensitivity (Fig. 4e,f). Glucose turnover, glycolysis and glycogen synthesis were similar in the *ob/ob/Fsp27*^−/−^ group (Fig. 4g). However, radio-isotope tracer analysis suggested that hepatic glucose output (production) was higher in the *ob/ob/Fsp27*^−/−^mice than in the *ob/ob* group in the basal state and during the insulin infusion (Fig. 4h). Consistently, expression levels of gluconeogenic genes (*G6pc* and *Pck1*) were increased in the liver of *ob/ob/Fsp27*^−/−^mice (Fig. 4i).

We next assessed insulin signalling in various tissues of *ob/ob/Fsp27*^−/−^ mice following tail vain injection of insulin. Insulin-induced Akt phosphorylation was similar in the muscle and BAT of *ob/ob* and *ob/ob/Fsp27*^−/−^mice, and was increased in the GWAT of *ob/ob/Fsp27*^−/−^ mice (Fig. 4j–l). However, insulin-stimulated Akt phosphorylation was significantly reduced in the liver of *ob/ob/Fsp27*^−/−^ mice (Fig. 4m), suggesting reduced hepatic insulin signalling. These data indicate that *ob/ob/Fsp27*^−/−^ mice have systemic insulin resistance, which is mostly attributed to severe hepatic insulin resistance despite reduced inflammation and increased insulin-stimulated Akt phosphorylation in WAT.

### Fatty liver and insulin resistance in HFD-fed Fsp27^−/−^ mice

**Figure 5:**
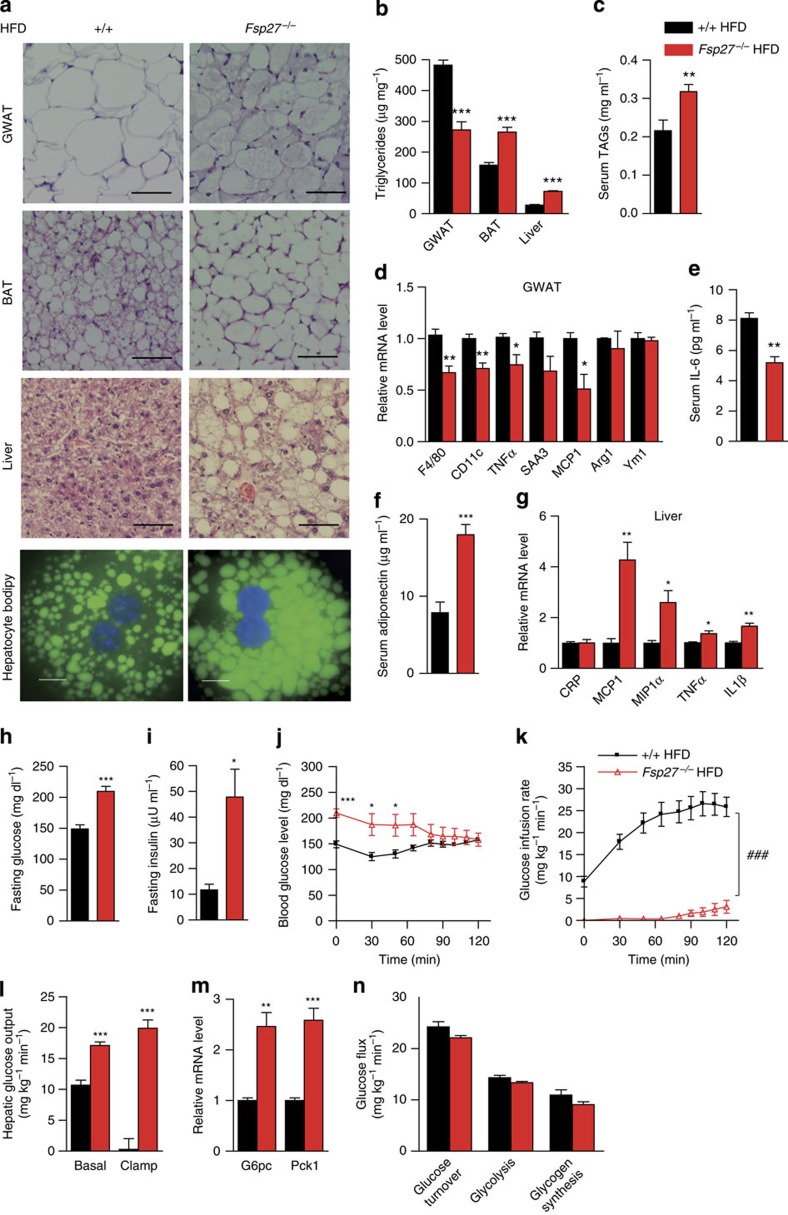
Hepatic steatosis and insulin resistance but reduced WAT inflammation in HFD-fed *Fsp27*^−/−^ mice. Three-month-old WT and *Fsp27*^−/−^ mice were challenged with a HFD (D12331, 58% kcal of fat) for 3 months (**a**–**g**). (**a**) Morphology of WAT, BAT and liver in WT and *Fsp27*^−/−^ mice. Scale bar, 64 μm for H&E (haematoxylin and eosin staining). Isolated hepatocytes were stained with bodipy 493/503. Scale bar, 10 μm. (**b**) TAG content in WAT, BAT and liver (*n*=5 per group). (**c**) Serum TAG concentrations (*n*=6 for WT and *n*=8 for *Fsp27*^−/−^). (**d**) Relative mRNA levels (*n*=4 per group). (**e**) Serum IL-6 concentrations (*n*=7 per group). (**f**) Serum adiponectin concentrations (*n*=8 per group). (**g**) Relative mRNA levels (*n*=4 per group). Three-month-old WT (*n*=6) and *Fsp27*^−/−^ mice (*n*=6) were challenged with an HFD (D12492, 60% kcal of fat) for 6 weeks (**h**–**n**). Fasting blood glucose (**h**) and insulin (**i**) concentrations of WT and *Fsp27*^−/−^ mice. Peripheral and hepatic insulin sensitivity were assessed using hyperinsulinaemic–euglycaemic clamps (**j**–**l**,**n**; WT, *n*=6; *Fsp27*^−/−^, *n*=6). (**j**) Blood glucose concentrations during the hyperinsulinaemic–euglycaemic clamp. (**k**) Glucose infusion rates. (**l**) Hepatic glucose output during the basal and hyperinsulinaemic clamp conditions. (**m**) Relative mRNA level of *G6pc* and *Pck1* in the liver (*n*=4 per group). (**n**) Peripheral glucose turnover. Quantitative data are presented as mean±s.e.m. Significance was established using a two-tailed Student’s *t*-test. Differences were considered significant at *P*<0.05.*P<0.05, **P<0.01, ***P<0.001. Two-way repeated-measurement analyses of variance were used to evaluate the data in Fig. 5k (^###^*P*<0.001 in this figure indicates that the two groups respond differently following the intervention).

To check whether reduced WAT inflammation and hepatic insulin resistance also occur in *Fsp27*-deficient mice with diet-induced obesity, *Fsp27*^−/−^ mice were fed with an high-fat diet (HFD; Research Diet, D12331) for 3 months. HFD feeding (for 3 months) has very little effect on Fsp27 expression in WAT depots in WT mice (Supplementary Fig. 4a). However, Fsp27 expression is clearly elevated in WAT depots in *ob/ob* mice (Supplementary Fig. 4a). Body weight remained similar in HFD-fed WT and *Fsp27*^−/−^ mice (Supplementary Fig. 4b,c), but liver and BAT mass were significantly higher in the *Fsp27*^−/−^ mice (Supplementary Fig. 4d,e). Fat mass, including GWAT, subcutaneous fat and mesenteric fat, was significantly reduced in *Fsp27*^−/−^ mice (Supplementary Fig. 4d,e). Reduced LD sizes and lower TAG levels were observed in the WAT of *Fsp27*^−/−^ mice (Fig. 5a,b). In contrast, serum TAG concentrations (Fig. 5c), the size of LDs and cellular TAG content in the BAT, liver and isolated hepatocytes of HFD-fed *Fsp27*^−/−^ mice were all increased (Fig. 5a,b). In agreement with reduced inflammation in the WAT of *ob/ob*/*Fsp27*^−/−^ mice, expression levels of *F4/80*, *TNFα* and *MCP1* in the WAT of HFD-fed *Fsp27*^−/−^ mice were reduced (Fig. 5d). Reduced serum IL-6 concentrations and elevated serum adiponectin concentrations were also observed in these mice (Fig. 5e,f). Consistent with increased hepatic inflammation in *ob/ob*/*Fsp27*^−/−^ mice, expression levels of *MCP1*, *MIP1α*, *TNFα* and *IL-1β* were increased in the liver of HFD-fed *Fsp27*^−/−^ mice (Fig. 5g). Thus, HFD-fed *Fsp27*-deficient mice displayed reduced WAT inflammation but increased hepatic lipid accumulation.

Next, we measured fasting glucose and insulin concentrations, and found them to be significantly higher than those of WT mice (Fig. 5h,i). Blood glucose concentrations were also higher in the HFD-fed *Fsp27*^−/−^ mice during the first 1 h of the clamp experiment (Fig. 5j). When infusing insulin at a constant rate (3 mU kg^−1^ min^−1^), the glucose infusion rate required to maintain euglycaemia was much lower in *Fsp27*^−/−^ mice fed with an HFD (Fig. 5k). In addition, basal and clamp hepatic glucose output were increased in the HFD-fed *Fsp27*^−/−^ group (Fig. 5l). Consistent with increased glucose output, the expression levels of *G6pc* and *Pck1* were increased in the liver of HFD-fed *Fsp27*^−/−^ mice (Fig. 5m). Glucose turnover, glycolysis and glycogen synthesis were similar in the HFD-fed *Fsp27*^−/−^ group (Fig. 5n). Overall, these data suggest that *Fsp27* deficiency also induces hepatic steatosis and insulin resistance despite reduced WAT inflammation in HFD-fed mice.

### Fatty liver and insulin resistance in BATless/Fsp27^−/−^ mice

**Figure 6:**
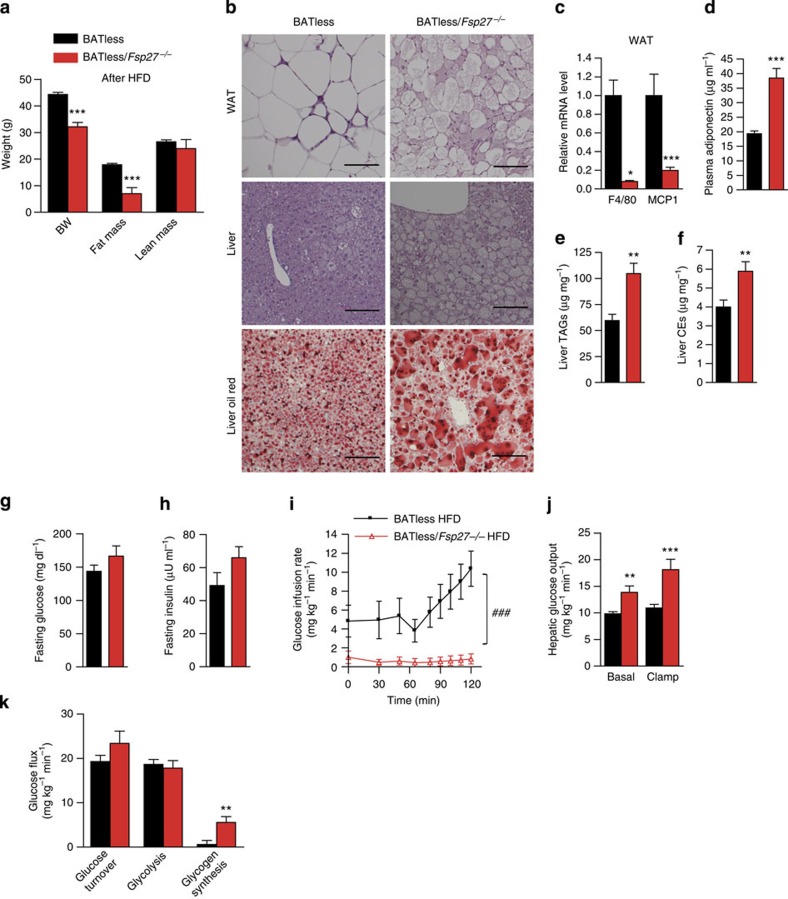
Reduced adipose inflammation but hepatic insulin resistance in BATless*/Fsp27*^−/−^ mice. Three-month-old BATless (*n*=12) and BATless*/Fsp27*^−/−^ (*n*=8) mice were challenged with HFD (D12492, 60% kcal of fat) for 6 weeks. (**a**) Body composition after the HFD. (**b**) Morphology of WAT (upper panel) and the liver (middle panel); Oil-red staining (lower panel) of the liver in BATless and BATless*/Fsp27*^−/−^mice. Scale bar, 64 μm for H&E (haematoxylin and eosin staining). (**c**) Relative mRNA levels of macrophage markers *F4/80* and *MCP1* in WAT (BATless, *n*=11; BATless*/Fsp27*^−/−^, *n*=7). (**d**) Plasma concentration of adiponectin (BATless, *n*=11; BATless*/Fsp27*^−/−^, *n*=8). (**e**) Liver TAG content and (**f**) liver CE content (BATless, *n*=9; BATless*/Fsp27*^−/−^, *n*=7). (**g**) Fasting glucose and (**h**) fasting insulin concentration in BATless and BATless*/Fsp27*^−/−^ mice. Peripheral and hepatic insulin sensitivity were assessed using hyperinsulinaemic–euglycaemic clamps (**i**–**k**). (**i**) Glucose infusion rates. (**j**) Hepatic glucose output during basal and hyperinsulinaemic clamp conditions. (**k**) Peripheral glucose turnover. Quantitative data are presented as mean±s.e.m. Significance was established using a two-tailed Student’s *t*-test. Differences were considered significant at *P*<0.05.*P<0.05, **P<0.01, ***P<0.001. Two-way repeated-measurement analyses of variance were used to evaluate the data in Fig. 6i (^###^*P*<0.001 in this figure indicates that the two groups respond differently following the intervention).

As BAT could conceivably aid adaptation to any deficiency in WAT lipid storage, especially in mice with less fat mass housed in a relatively cold environment where thermogenesis is typically enhanced, we also sought to ascertain the importance of thermogenic BAT in these mice by crossing the *Fsp27* knockouts with BATless mice^23^. BATless mice were previously shown to lack BAT as a result of BAT-selective expression of diphtheriatoxin and are susceptible to HFD-induced obesity and insulin resistance^28^. At age 3 months, BATless and littermate BATless/*Fsp27*^−/−^ mice were placed on an HFD (Research Diet, D12492) for 6 weeks before analysis at age 4.5 months. Body weight and fat mass were significantly lower in the BATless/*Fsp27*^−/−^ mice after HFD feeding than in the BATless group, whereas lean mass was similar (Fig. 6a and Table 2). WAT morphology in the BATless/*Fsp27*^−/−^ mice showed similar changes to those observed in the *ob/ob/Fsp27*^−/−^ and HFD-fed *Fsp27*^−/−^ models (Fig. 6b). The expression level of *F4/80* and *MCP1* was significantly lower in the WAT of BATless/*Fsp27*^−/−^ mice (Fig. 6c), and in keeping with our observations in the *ob/ob/Fsp27*^−/−^ mice and HFD-fed *Fsp27*^−/−^ mice we again noted higher plasma adiponectin levels in the BATless/*Fsp27*^−/−^ mice (Fig. 6d). Liver TAG and CE levels were higher in the BATless/*Fsp27*^−/−^ mice than in the BATless mice (Fig. 6b,e,f). These data indicate that BATless/*Fsp27*^−/−^ mice have reduced WAT inflammation but increased hepatic lipid storage.

Despite similar concentrations of fasting glucose and insulin in BATless and BATless/*Fsp27*^−/−^ mice (Fig. 6g,h), the glucose infusion rate was again significantly lower in the BATless/*Fsp27*^−/−^ group (Fig. 6i), suggesting reduced insulin sensitivity. Similar to the observations in the *ob/ob/Fsp27*^−/−^ and the HFD-fed *Fsp27*^−/−^ mice, both basal and clamp hepatic glucose production were increased in the BATless/*Fsp27*^−/−^ mice, suggesting significant hepatic insulin resistance (Fig. 6j). Glucose turnover and glycolysis rates were similar, whereas glycogen synthesis appeared to be elevated in the BATless/*Fsp27*^−/−^ mice (Fig. 6k). In conclusion, *Fsp27* deficiency in this BAT-deficient model also resulted in reduced WAT inflammation but increased hepatic insulin resistance.

### Increased Cidea expression in ob/ob/Fsp27^−/−^ mice liver

**Figure 7:**
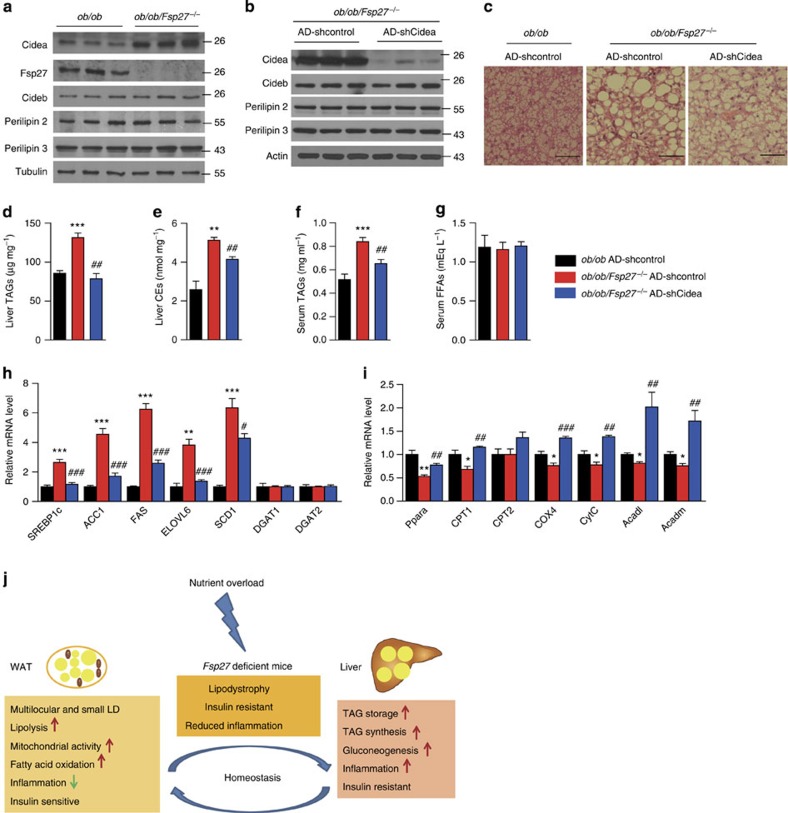
Liver-specific knockdown of Cidea alleviates hepatic stestosis in *ob/ob/Fsp27*^−/−^ mice. Four-month-old chow-fed *ob/ob* and *ob/ob/Fsp27*^−/−^ mice were used to generate these data (**a**). Four-month-old chow-fed *ob/ob* or *ob/ob/Fsp27*^−/−^ mice were injected with AD-shcontrol or AD-Cidea for 1 week before analysis (**b**–**i**). (**a**) Expression of the indicated proteins in the liver of *ob/ob* and *ob/ob/Fsp27*^−/−^ mice. (**b**) Protein expression in the liver of *ob/ob/Fsp27*^−/−^ mice injected with the indicated adenoviral vectors. (**c**) Liver H&E (haematoxylin and eosin) staining of *ob/ob/Fsp27*^−/−^ mice injected with AD-shcontrol or AD-shCidea adenoviral vectors. Scale bar, 64 μm. (**d**) Liver TAG and (**e**) CE concentration in the livers of *ob/ob* and *ob/ob/Fsp27*^−/−^ mice injected with the indicated adenovirusus (*n*=4). (**f**) Serum free fatty acid (*n*=7) and (**g**) TAG concentrations (*n*=6). (**h**,**i**) Relative mRNA expression levels in the livers of adenovirus injected *ob/ob* and *ob/ob/Fsp27*^−/−^ mice (*n*=4 per group). (**j**) Proposed model for the role of Fsp27 in regulating metabolism. Quantitative data are presented as mean±s.e.m. Significance was established using a two-tailed Student’s *t*-test. Differences were considered significant at *P*<0.05.*P<0.05, **P<0.01, ***P<0.001.

As mentioned previously, Fsp27 expression is typically increased in steatotic livers and knocking it down has been shown to alleviate hepatic steatosis^5^. We therefore sort to understand the molecular pathways involved in mediating the hepatic steatosis observed in our *Fsp27*^−/−^ models. We began by checking the expression levels of several LD-associated proteins in the livers of *ob/ob* and *ob/ob/Fsp27*^−/−^ mice. Cidea expression (mRNA and protein) was markedly increased in the livers of *ob/ob/Fsp27*^−/−^ mice (Fig. 7a (protein) and Supplementary Fig. 5a (mRNA)), whereas expression of other LD-associated proteins including Cideb and Perilipin 2/3 were similar between *ob/ob/Fsp27*^−/−^ and *ob/ob* mice (Fig. 7a and Supplementary Fig. 5a). The protein stability of Cidea but not Cideb and Perilipin 2 was also significantly increased in the liver of *ob/ob/Fsp27*^−/−^ mice, suggesting that the observed increase in Cidea expression is a consequence of transcriptional changes as well as changes in protein degradation (Supplementary Fig. 5b). Similar changes in Cidea protein levels were apparent in the livers of HFD-fed *Fsp27*^−/−^ mice (Supplementary Fig. 5c–e).

To evaluate the physiological significance of the observed changes in hepatic Cidea expression, we proceeded to knockdown *Cidea* expression using adenovirally delivered short hairpin RNA against Cidea in *ob/ob/Fsp27*^−/−^ mice. This strategy resulted in a substantial (~90%) knockdown of Cidea (Fig. 7b) but had no discernible effect on Cideb or Perilipin2/3 expression (Fig. 7b). Depletion of Cidea led to a significant reduction in LD size and both TAG and CE content in the liver of *ob/ob/Fsp27*^−/−^ mice (Fig. 7c–e). Knocking down *Cidea* in isolated hepatocytes from *ob/ob/Fsp27*^−/−^ or HFD-fed *Fsp27*^−/−^ mice also reduced TAG concentration (Supplementary Fig. 5f–i). These changes in liver lipid accumulation were not associated with differences in food intake, body weight or serum free fatty acid concentrations (Fig. 7f). However, they did correspond with differences in serum TAG concentrations (Fig. 7g).

These findings suggest that the observed increase in hepatic Cidea expression compensates for the absence of Fsp27 and facilitates liver lipid accumulation. Furthermore, many of the changes in gene expression noted in the livers of *ob*/*ob*/*Fsp27*^−/−^ mice (see Fig. 3i–k) were reversed following *Cidea* knockdown. Specifically, expression levels of SREBP1c and some of its downstream target genes (*ACC1*, *FAS*, *Elovl6* and *SCD1*) were significantly reduced when *Cidea* was depleted in the liver of *ob*/*ob*/*Fsp27*^−/−^ mice (Fig. 7h), whereas expression of *Ppara* and some of its target genes were significantly increased in the liver of these mice (Fig. 7i). Collectively, these data suggest that Cidea compensates functionally for the absence of Fsp27 in regulating hepatic lipid storage in ‘obese’ *Fsp27*-deficient mice.

## Discussion

In summary, our data suggest that *Fsp27* deficiency significantly reduces the capacity of white adipocytes/adipose tissue to store lipids in the face of severe energy overload and thus enhances susceptibility to develop liver steatosis and hepatic insulin resistance (schematically illustrated in Fig. 7j). In this context, increases in hepatic Cidea expression appear to compensate for the absence of liver Fsp27 expression and contribute to the development of hepatic steatosis. These mouse data are consistent with the lipodystrophic phenotype reported in the human patient with a homozygous CIDEC mutation^14^. Although this defect in lipid storage is at least partly compensated for by increased fat oxidation and energy expenditure, the very modest increase in energy expenditure is not sufficient to dispose off all the excess lipid/energy delivered to WAT in the context of *leptin* deficiency or long-term exposure to an HFD; thus, lipid starts to accumulate in the liver. Here it impairs insulin signalling and induces hepatic insulin resistance, which manifests as increased hepatic glucose output and increased lipogenesis. Exactly how liver steatosis leads to insulin resistance has been the subject of many previous studies^29^ but remains incompletely understood^30^.

Our findings suggest that in the context of surplus energy intake, *Fsp27* deficiency can switch a relatively ‘lean and healthy’ insulin-sensitive animal to a ‘lipodystrophic’ insulin-resistant state. This is particularly important when considering the lean phenotypes observed in several other mouse models and their potential relevance to humans, as mice are conventionally fed a low-fat diet and housed at a sub-thermoneutral temperature, conditions in which mice require higher metabolic activity, whereas humans live in a thermoneutral environment and consume diets typically containing considerably more fat over prolonged periods of time. It is thus tempting to speculate that other ‘lean’ mouse models^19^,^22^,^31^,^32^ that arise from defects in neutral lipid storage in WAT may also manifest a lipodystrophic phenotype with the development of hepatic steatosis and insulin resistance when exposed to a more extreme nutritional challenge as was previously reported for mice with a loss-of-function *Pparg* mutation^33^.

Our conclusions differ from those previously reported by Nishino *et al*.^13^, who reported improved whole body and hepatic insulin sensitivity in 12-week-old, HFD-fed (from age 4 weeks) *Fsp27*-null mice, and from those of Toh *et al*.^12^, who reported improved glucose and insulin tolerance in 10-week-old chow-fed *ob/ob/Fsp27*^−/−^. To comprehensively address these discrepancies, we have studied both of these independently generated *Fsp27* knockout lines, involved collaborators from both of these original studies and ‘nutritionally’ challenged the *Fsp27* knockouts in three different ways. The consistency of our current findings is in our view compelling evidence for the conclusions we report. The precise reasons for the discrepancies between our data and those of Nishino *et al*.^13^ are difficult to be certain of; all we can say is that they studied the mice at a younger age (~12 versus ~20 weeks) and on a different HFD formulation, in a different laboratory environment. The work reported by Toh *et al*.^12^ did not include hyperinsulinaemic-euglycaemic clamps and the mice were again studied at a younger age (~10 weeks) than in the current studies. The differences we observed in glucose tolerance tests and ITTs were still relatively subtle in the older mice we studied; hence, this might explain, at least in part, the discrepancy.

One of the most striking observations in the *ob/ob/Fsp27*^−/−^, HFD-fed *Fsp27*^−/−^ mice and BATless/Fsp27^−/−^ mice was the disassociation of WAT inflammation and insulin resistance. We had anticipated that ‘overloading’ the *Fsp27*-deficient adipocytes would lead to an adverse WAT phenotype. Instead, the accumulation of smaller multilocular LD containing adipocytes was associated with reduced inflammasome activation, less adipocyte death, and hence fewer CLSs and a reduction in WAT inflammatory cytokine expression. In addition, less systemic inflammation was demonstrated by the reduced serum TNFα and IL-6 levels, and increased adiponectin level was observed (Fig. 2). Consistent with these data, insulin stimulated Akt phosphorylation was improved in the WAT of *ob/ob/Fsp27*^−/−^ mice. Insulin-stimulated Akt phosphorylation in skeletal muscle was not affected by *Fsp2*7 deficiency. Glucose tolerance, one useful indicator of β-cell function was also unaffected by *Fsp2*7 deficiency. This represents a very rare, but informative, model of ‘pure WAT lipid storage limitation’ resulting in hepatic steatosis and hepatic insulin resistance; it also reminds us that although abundant data now emphasizes the importance of endocrine and cytokine WAT dysfunction, the primary role of WAT remains energy storage. Our data suggest that treating WAT inflammation alone may not be sufficient to improve systemic insulin sensitivity unless energy balance is also favourably modified. Clearly, our data does not prove that anti-inflammatory approaches to treating obesity-associated insulin resistance will not be useful and we are aware of several mouse studies in which knockdown or inhibition of inflammatory signalling intermediates were shown to improve insulin sensitivity^34^,^35^. Nevertheless, our data at least suggest that this may be a contributing factor in explaining the relatively modest benefits observed to date in response to anti-inflammatory therapies in humans with type 2 diabetes^36^,^37^.

## Methods

### Animal models

The double knockout mice *ob/ob/Fsp27*^−/−^ were generated by crossing *Fsp27*^−/−^ mice generated by Toh *et al*.^12^ with *leptin*^*+/*−^ mice. Mice used were on a C57BL/6J background. Male mice were studied in all cases. Mice were fed with a chow diet (ND, 5053, PicoLab Rodent Diet20, Research Diet, USA) or an HFD (D12331, Research Diet). For the HFD experiments presented in Fig. 5a–g, Supplementary Fig. 4a–e and Supplementary Fig. 5c–e,h,i, 3-month-old mice were provided with an HFD for 3 months. Mouse experiments were performed in the animal facility of the Center of Biomedical Analysis at Tsinghua University (Beijing, China). The laboratory animal facility has been accredited by the AAALAC (Association for Assessment and Accreditation of Laboratory Animal Care International), and the IACUC (Institutional Animal Care and Use Committee) of Tsinghua University approved all animal protocols used in this study. Computed tomography (CT) analysis was performed using the Latheta LCT-200, Hitachi Aloka, Japan. Magnetic resonance imaging analysis was performed at the Department of Radiology, Southeast University, Nanjing, China^38^.

BATless transgenic mice (FVB/N-Tg(UcpDta)1Kz/J) were purchased from The Jackson Laboratory (Bar Harbor, Maine) and crossed with *Fsp27*^−/−^ mice generated by Nishino *et al*.^13^, to generate BATless/*Fsp27*^−/−^ mice. These mice were rederived onto a C57BL/6J background and interbred thereafter. At age 3 months, BATless and littermate BATless/*Fsp27*^−/−^ mice were placed on an HFD (D12492) for 6 weeks before surgery, recovery and then hyperinsulinaemic–euglycaemic clamps at age 4.5 months. Three-month-old WT and *Fsp27*^−/−^ mice generated by Toh *et al*.^12^ were placed on an HFD (Research Diet, D12492) for 6 weeks before surgery, recovery and then hyperinsulinaemic–euglycaemic clamps at age 4.5 months. The BATless mouse studies and all hyperinsulinaemic–euglycaemic clamps were accredited by the AAALAC. The IACUC of Center of Animal Care and Use at Lee Gil Ya Cancer and Diabetes Institute, Gachon University (Incheon, Korea) approved all animal protocols used in this project (approval number: DI-2011-0044 and LCDI-2013-0053).

### Generation and administration of recombinant adenoviruses

Recombinant adenoviruses used for the knockdown of Cidea (AD-shCidea) and control (AD-shcontrol) were constructed using the AdEasy Adenoviral Vector System (Stratagene, USA). The short hairpin RNA targeting sequence of Cidea was as follows: 5′- ACACGCATTTCATGATCTT -3′. The recombinant adenoviruses were produced and purified according to the manufacturer’s instructions. Following a large-scale amplification in AD293 cells and CsCl adenoviral purification, the titres of the adenoviruses were determined using the AdEasy Viral Titer Kit (Stratagene). The viruses were stored at −80 °C. For the *in vivo* infection, 4-month-old mice were intravenously injected in the tail vein with 1 × 10^10^ viral particles of the indicated viruses in a total volume of 200 μl and were euthanized for tissue collection 7 days later. For the infection of isolated primary hepatocytes, the cells were infected with the indicated viruses in serum-free DMEM for 4 h, followed by the addition of fetal bovine serum (FBS) to a final concentration of 10%. Twenty-four hours later, the cells were harvested for further experiments.

### Isolation of primary hepatocytes

Mouse primary hepatocytes were isolated as follows. Four-month-old male *ob/ob* or *ob/ob/Fsp27*^−/−^ mice were anaesthetized with 1% Pelltobarbitalum Natricum (Amresco, USA) before exposing the hepatic portal veins, which were washed to remove residual blood and then perfused with collagenase (C5138, Sigma, USA) for about 10 min. Thereafter, the livers were immediately moved to a sterile 10-cm cell culture dish for mincing before the hepatocytes were dispersed, by aspiration with a large-bore pipette; the hepatocytes were then filtered through a 70-μm membrane (Millipore, USA) to remove tissue debris. After washing twice with cold DMEM and centrifuging at 50*g* for 4 min at 4 °C, the isolated hepatocytes were seeded at a density of 1 × 10^7^ cells per dish in 6-cm dishes in DMEM with 10% FBS. The medium was changed 6 h after seeding. Isolated hepatocytes were maintained in DMEM (Invitrogen, USA) containing 10% FBS (Invitrogen). For protein stability experiment, medium was replaced with fresh DMEM plus 10% FBS and Cycloheximide (100 μg ml^−1^, Sigma). Hepatocytes were harvested at different time points after the addition of Cycloheximide.

### Serum and plasma biochemical and metabolic analyses

Serum TAG concentrations were measured using Serum Triglyceride Determination Kit (Sigma) following the manufacturer’s instructions. Plasma glycerol concentrations were determined using the Free Glycerol Reagent (Sigma). The free fatty acid concentrations were determined using enzymatic assay kits (Wako Pure Chem, Japan). The serum concentrations of IL-6 and TNFα were determined using the Mouse IL6 Elisa Ready-SET-GO kit and Mouse TNFα Elisa Ready-SET-GO kit (eBioscience, USA). Serum concentrations of adiponectin were determined using the enzymatic methods (Abcam, USA, ab108785). Plasma concentrations of adiponectin in BATless background mice were determined using the Adiponectin Elisa kit, 47-ADPMS-E01 (Alpco). Blood insulin concentrations were measured using a Rat Insulin RIA kit (Millipore, RI-13K).

### Mouse metabolic studies

Fat and lean body masses were measured by ^1^H minispec system (LF90II, Bruker Optik, Germany) in mice. Energy expenditure was determined using a MM-100 Metabolic Monitor system (CWE, Inc., USA). Experiments were performed on 4-month-old *ob/ob* and *ob/ob/Fsp27*^−/−^ male mice. The mice were monitored individually in the Oxymax chamber for 24 h and were allowed to acclimate the chamber for several hours before commencing data collection. For HFD-fed mice, energy balance was assessed in a metabolic monitoring system (CLAMS, Columbus Instruments, USA) for 4 days (2 days of acclimation followed by 2 days of measurement).

### GTTs and ITTs

Glucose tolerance tests of *ob/ob* and *ob/ob/Fsp27*^−/−^ mice were performed in overnight-fasted mice following an intraperitoneal injection of glucose (0.5 g per kg body mass). ITTs of *ob/ob* and *ob/ob/Fsp27*^−/−^ mice were performed after an intraperitoneal injection of insulin (2 U per kg body mass) following a 6 h fast. Blood glucose concentrations were measured with glucose analyser (GM9, Analox Instruments Ltd, UK). For examination of *in vivo* insulin signalling, 4-month-old male *ob/ob* and *ob/ob/Fsp27*^−/−^ mice were fasted for 2 h, anaesthetized and injected with insulin (5 U per kg body weight). After 5 min, mice were killed and tissues were collected.

### Hyperinsulinaemic–euglycaemic clamp

After an overnight fast, [3-^3^H]-glucose (HPLC purified; American Radiolabeled Chemicals, USA) was infused at a rate of 0.05 μCi min^−1^ for 2 h, to assess the basal glucose turnover. Following the basal period, a hyperinsulinaemic–euglycaemic clamp was conducted for 120 min with a primed/continuous infusion of human insulin (Eli Lilly) (for BATless background mice and HFD-fed mice: 21 mU kg^−1^ during priming and 3 mU kg^−1^ min^−1^ during infusion; for *ob/ob* background mice: 105 mU kg^−1^ during priming and 15 mU kg^−1^ min^−1^ during infusion, whereas plasma glucose was maintained at basal concentrations (~180 mg dl^−1^ for *ob/ob* background mice; ~150 mg dl^−1^ for BATless background mice and HFD-fed mice))^39^. To estimate insulin-stimulated whole-body glucose fluxes, [3-^3^H]-glucose was infused at a rate of 0.1 μCi min^−1^ throughout the clamps^40^. Rates of basal and insulin-stimulated whole-body glucose fluxes and tissue glucose uptake were determined as previously described^40^.

### Microarray analysis

Equal amounts of total RNA from three mice were combined to form RNA pools. In total, we analysed three RNA pools from nine *ob/ob* mice and three RNA pools from nine *ob/ob/Fsp27*^−/−^ mice. Six Affymetrix gene chips (GeneChip Mouse Gene 1.0 ST Array, Affymetrix, USA) were used for hybridization and data collection. Microarray data related to WT and *Fsp27*^−/−^ mice were from Li *et al*. (GSE22693)^41^. Quality control and statistical analyses of all the Mouse Gene 1.0 ST microarray data was conducted using R/Bioconductor^42^. Methods including scatterplots, distribution histograms, boxplots and unsupervised Principle Component Analysis were employed to visualize the data before and after preprocessing procedures. All arrays were consistent and comparable for further analyses, and we performed background adjustment, quantile normalization and summaries of transcript-level intensity for all arrays using the Robust Multi-array Average algorithm^43^, followed by two rounds of probeset filtering. After removing control probesets, 28,858 probesets from the original 35,556 were retained. Next, the Detection Above Background *P*-values for probesets were calculated using the *xps* package and only the significant ones (*P*<0.05) were considered as ‘present’. We only retained probesets flagged as present in at least one sample for each type of tissue and used the package LIMMA to identify probesets that were differentially expressed between the *ob/ob/Fsp27*^−/−^ and *ob/ob* mice^44^. The Benjamini and Hochberg method was used to estimate the false discovery rate and correct for multiple hypotheses testing. Annotation was taken and genes that changed by log fold of at least 0.5 between *ob/ob/Fsp27*^−/−^ and *ob/ob* mice, and with a false discovery rate <0.05 were considered significant. The up- and downregulated genes were further mapped to biological pathways using PathVisio with Wiki Pathways content, and the results were sorted by *Z*-score, which is the standard statistical test under the hypergeometric distribution^45^,^46^.

### Quantitative PCR analysis

Total RNA was isolated from mouse livers and WAT with TRIzol (Invitrogen) extraction. First-strand complementary DNA synthesis was performed using the Superscript First-Strand Synthesis System (Invitrogen). Quantitative real-time PCR reaction were performed using the Power SYBR Green PCR Master Mix (Applied Biosystems) on an ABI 7500 (Applied Biosystems) with reaction volumes of 20 μl. The primer sequences are listed in Supplementary Table 3.

### Histology

The livers were excised and fixed in 10% formalin buffer. The fixed specimens were processed to paraffin blocks, sectioned and stained with haematoxylin–eosin. For electron microscope analysis, liver or fat tissue was fixed in 2.5% glutaraldehyde buffer and studied at Center of Biomedical Analysis, Tsinghua University, China. For immunohistochemistry, formalin-fixed and paraffin-embedded sections were blocked with endogenous peroxidase (3% H_2_O_2_ in 80% methanol) for 20 min. Antigen retrieval was performed in 10 mM sodium citrate in a microwave for 15 min. After blocking nonspecific antigen with normal goat serum for 30 min, the slides were then incubated with TNFα (Abcam, ab1793, 1:100 dilution) or F4/80 (Abcam, ab6640, 1:100 dilution) antibody overnight at 4 °C. The slides were then incubated with biotinylated-labelled secondary antibodies (1:200, GE Health, UK) for 30 min at room temperature. Visualization was performed using 0.1% 3,3'-diaminobenzidine (Dako, Denmark) in PBS together with 0.05% H_2_O_2_ (ref. 47). For the analysis of CLS, immunohistochemistry sections stained with a TNFα antibody were used and the CLS density was derived by counting the total number of CLS in each section compared with the total number of adipocytes. Data were expressed as the number of CLS/1,000 adipocytes.

### Tissue lipid content

Tissues were homogenized in PBS buffer with protease inhibitors. A chloroform/methanol (2:1) solution was rapidly added to the homogenate and the samples were vortexed. The samples were centrifuged at 250*g* for 10 min, to separate the phases. The lower lipid-containing phase was carefully aspirated and allowed to dry in a 70 °C metal bath with nitrogen steam. The dried lipids were reconstituted in methylbenzene and loaded onto a thin-layer chromatography plate. The lipids were separated in a hexane/diethyl ether/acetic acid (70:30:1, v/v) solution. The thin-layer chromatography plates were sprayed with 10% CuSO_4_ in 10% phosphoric acid and were developed by drying in an oven at 120 °C. Alternatively, the dried lipids were emulsified in chloroform with 5% Triton X-100. Finally, dried emulsified lipids with nitrogen gas were reconstituted in distilled water. The contents of TAG and CE were measured by enzymatic reaction according to the instruction manual (Wako Diagnotics, Japan).

### Western blotting

The frozen tissues were homogenized in a lysis buffer (20 mM Tris-HCl, 150 mM NaCl, 1 mM EDTA, 1 mM EGTA, 1% Triton-X100 and protease inhibitor, pH 7.4) and then centrifuged for 20 min at 10,000*g* to discard cell debris. The total protein concentrations were determined using a Bio-Rad kit (USA). The proteins were subjected to western blot analysis with the desired antibodies. The antibodies against Cidea (1:1,000), Cideb (1:500) and Fsp27 (1:2,000) were generated by injection of rabbits with His-tagged truncated Cidea (aa 1–195) and Cideb (aa 1–176), and Fsp27 (aa 1–190) proteins that were expressed in and purified in *Escherichia coli*^12^. Antibodies against β-actin (Sigma-Aldrich, USA, 1:2,000), Perilipin1 (Fitzgerald Industries, USA, 20R-pp004, 1:4,000), Perilipin2 (Fitzgerald Industries, 20R-Ap002, 1:8,000), Perilipin3 (Santa Cruz, USA, sc14726R, 1:1,000), Cyto C (BD Pharmingen, USA, 556433, 1:1,000), Cox4 (Molecular Probes, Invitrogen, A21348, 1:1,000), ATGL (Cell Signaling, USA, 2439, 1:1,000), HSL (Cell Signaling, 4107, 1:1,000), CGI58 (Santa Cruz, sc100468, 1:1,000), PPARγ (Santa Cruz, sc271392, 1:1,000), CEBPβ (Santa Cruz, sc7962, 1:1,000), P-AKT(S473,193H12, Cell Signaling, 4058, 1:1,000), AKT (Cell Signaling, 9272, 1:1,000) were used for western blot analysis. The blots were developed using HRP-conjugated secondary antibodies (GE Health, 1:3,000) and the ECL-plus system.

### Statistics

The statistical data reported includes results from at least three biological replicates. All results are expressed as mean±s.e.m.. All statistical analyses were performed in GraphPad Prism Version 5 (GraphPad Software). Significance was predominantly established using a two-tailed Student’s *t*-test. However, energy expenditure was analysed using analysis of covariance as recommended by Tschop *et al*.^48^ and we used two-way repeated-measurement analyses of variance to evaluate the data in Figs 4c,d,f, 5k and 6i. (^###^*P*<0.001 in these figures indicates that the two groups respond differently following the intervention). In all cases, differences were considered significant at *P*<0.05. *P*-values are indicated in each figure as *P<0.05, **P<0.01, ***P<0.001.

## Author contributions

L.Z., S.P., L.X., C.C., D.S. and P.L. conceived and designed the experiments. L.Z., S.P., L.X., K.J., J.H., H.O., M.Y., L.S. and L.W. performed the experiments. L.Z., S.P., C.C., X.Y., D.S. and P.L. Analyzed the data. J.Y., Y.T., C.Z., S.C., J.A., S.J., F.G., and H.Y. contributed reagents/materials/analysis tools. L.Z., P.L. and D.S. wrote the paper.

## Additional information

**How to cite this article:** Zhou, L. *et al*. Insulin resistance and white adipose tissue inflammation are uncoupled in energetically challenged Fsp27-deficient mice. *Nat. Commun.* 6:5949 doi: 10.1038/ncomms6949 (2015).

**Accession codes:** The Gene Expression omnibus (GEO) accession number for the gene expression data is GSE59807.

## Supplementary Material

Supplementary Information Supplementary Figures 1-8 and Supplementary Tables 1-3

## Figures and Tables

**Table 1:** Tissue weights and blood biochemistry of *ob/ob* and *ob/ob/Fsp27* ^−/−^ mice.

**Parameter**	***ob/ob***		***ob/ob/Fsp27***^**−/−**^		
	***N***	**Mean±s.e.m.**	***N***	**Mean±s.e.m.**	***P*****-value**
Body weight (g)	10	52.09±1.13	10	36.82±0.33	<0.0001***
Serum glycerol (mg ml^−1^)	9	0.76±0.08	7	1.06±0.04	0.0092**
Serum NEFA (mEq l^−1^)	7	1.19±0.15	9	1.15±0.10	0.8376
Gonadal fat (g)	9	2.08±0.19	8	0.18±0.01	<0.0001***
Subcuteneous fat (g)	9	2.34±0.11	8	0.47±0.04	<0.0001***
Mesenteric fat (g)	9	1.64±0.06	8	0.34±0.01	<0.0001***
Retroperitoneal fat (g)	9	2.25±0.12	8	0.46±0.02	<0.0001***
Inguinal fat (g)	9	0.50±0.05	8	0.08±0.01	<0.0001***
Muscle(gastrocnemius) (g)	10	0.45±0.01	10	0.45±0.01	0.886
Kidney (g)	10	0.45±0.01	10	0.46±0.01	0.250
Heart (g)	10	0.16±0.002	10	0.16±0.003	0.307
Spleen (g)	10	0.10±0.006	10	0.11±0.003	0.398
Liver (g)	6	3.49±0.19	10	5.06±0.27	<0.001***
BAT (g)	5	0.39±0.03	7	0.70±0.06	<0.01**

BAT, brown adipose tissue; *N*, the number of mice used; NEFA, non-esterified fatty acid.

Four-month-old chow fed *ob/ob* and *ob/ob/Fsp27*^−/−^ mice were used. Quantitative data are presented as mean±s.e.m. Significance was established using a 2-tailed Student’s *t*-test. Differences were considered significant at *P*<0.05. **P<0.01, ***P<0.001.

**Table 2:** Body composition and tissue weights of BATless and BATless*/Fsp27* ^−/−^ mice while on a HFD.

**Parameter**	**BATless**		**BATless/Fsp27**^**−/−**^		***P* value**
	***N***	**Mean±s.e.m.**	***N***	**Mean±s.e.m.**	
Body weight (g)	12	44.3±0.8	10	32.2±1.6	<0.0001***
Fat mass (g)	12	17.9±0.5	10	7.0±2.3	<0.0001***
Lean mass (g)	12	26.5±0.7	10	24.0±3.4	0.2272
Liver (g)	12	1.4±0.08	10	1.7±0.07	0.0071**
Gonadal fat (g)	12	1.4±0.05	10	0.149±0.02	<0.0001***
BAT (mg)	12	35.4±3.2	10	66.7±16.4	0.0445*
Heart (mg)	12	136.4±3.0	10	144.6±4.0	0.1104
Gastrocnemius (mg)	12	160.9±2.8	10	172.1±4.2	0.0342*
Tibialis anterior (mg)	12	45.6±2.6	10	57.1±1.5	0.0017**
Quadriceps (mg)	12	148.5±7.3	10	183.5±9.2	0.0069**

HFD, high fat diet; N, the number of mice used.

Three-month-old BATless and BATless/*Fsp27*^−/−^ mice were challenged with a HFD (D12492, 60% kcal of fat) for 6 weeks. Quantitative data are presented as mean±s.e.m. Significance was established using a two-tailed Student’s *t*-test. Differences were considered significant at *P*<0.05. *P<0.05, **P<0.01, ***P<0.001.
