# Supplementary material for: Insulin resistance and white adipose tissue inflammation are uncoupled in energetically challenged Fsp27-deficient mice
Source: Nat Commun. 2015 Jan 7;6:5949. doi: 10.1038/ncomms6949 (PMC4354252; doi:10.1038/ncomms6949)
Supplement: Supplementary Information — Supplementary Figures 1-8 and Supplementary Tables 1-3 [file ncomms6949-s1.pdf]

Supplementary Figure 1

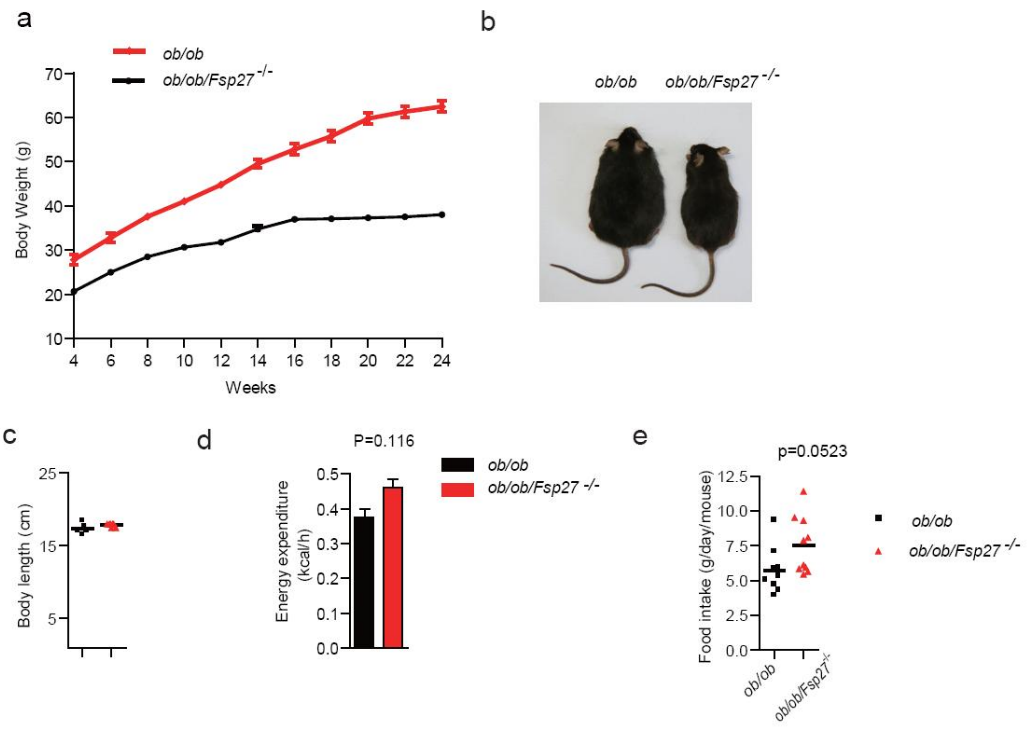

Supplementary Figure 1: Food intake and energy expenditure of *ob/ob* and *ob/ob/Fsp27$^{-/-}$* mice. 4 month old chow fed *ob/ob* and *ob/ob/Fsp27$^{-/-}$* mice were used for the analyses represented in panels b-e. (a) Growth curves demonstrating changes in body weight of *ob/ob* and *ob/ob/Fsp27$^{-/-}$* mice (n=4). (b) Representative photographs of an *ob/ob* and *ob/ob/Fsp27$^{-/-}$* mouse. (c) Body length of the *ob/ob* (n=5) and *ob/ob/Fsp27$^{-/-}$* (n=7) mice. (d) Energy expenditure of the *ob/ob* and *ob/ob/Fsp27$^{-/-}$* mice (n=4 per group). (e) Food intake of *ob/ob* (n=9) and *ob/ob/Fsp27$^{-/-}$* (n=10) mice. Quantitative data are presented as mean ± SEM. Significance was determined using a 2-tailed Student's t test.

Supplementary Figure 2

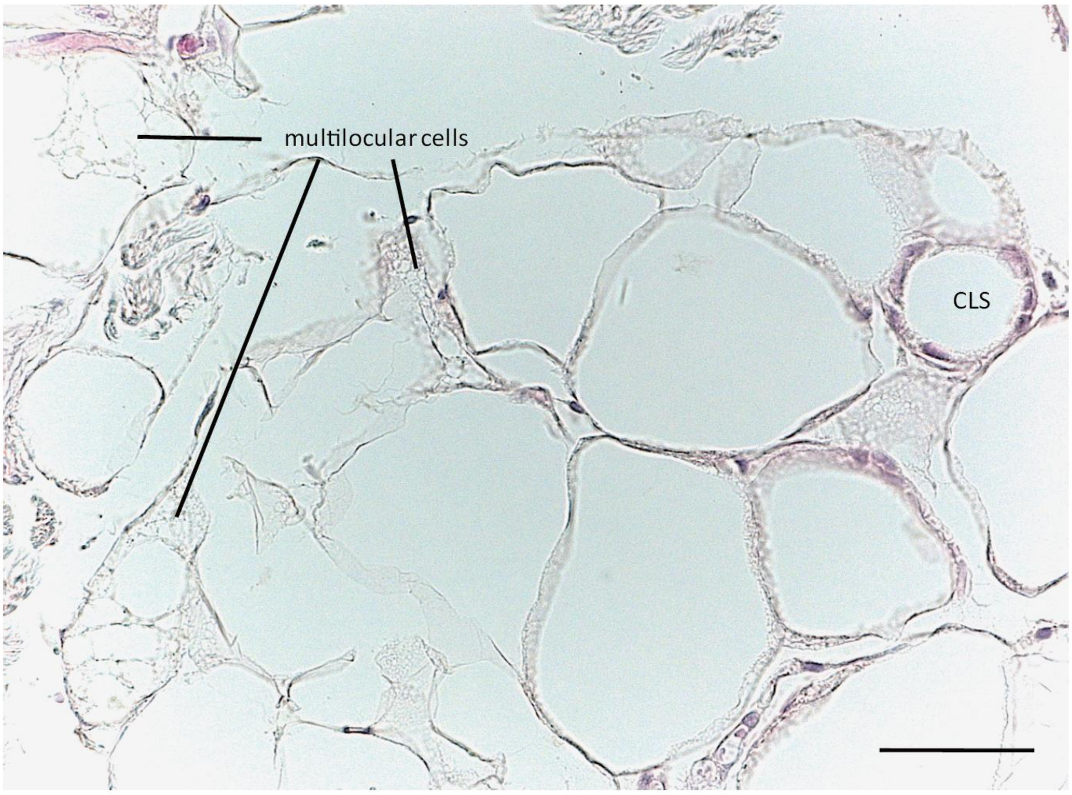

Supplementary Figure 2: Crown-like structures in white adipose tissue of the CIDEC E186X patient. An axilliary fat sample from a patient previously reported to have a homozygous loss-of-function mutation (E186X) in CIDEC. Histological analysis shows a mixed population of uni- and multi-locular (highlighted with black arrows) white adipocytes with a single crown-like structure (CLS) in this section. Scale bar= 50 μm.

Supplementary Figure 3

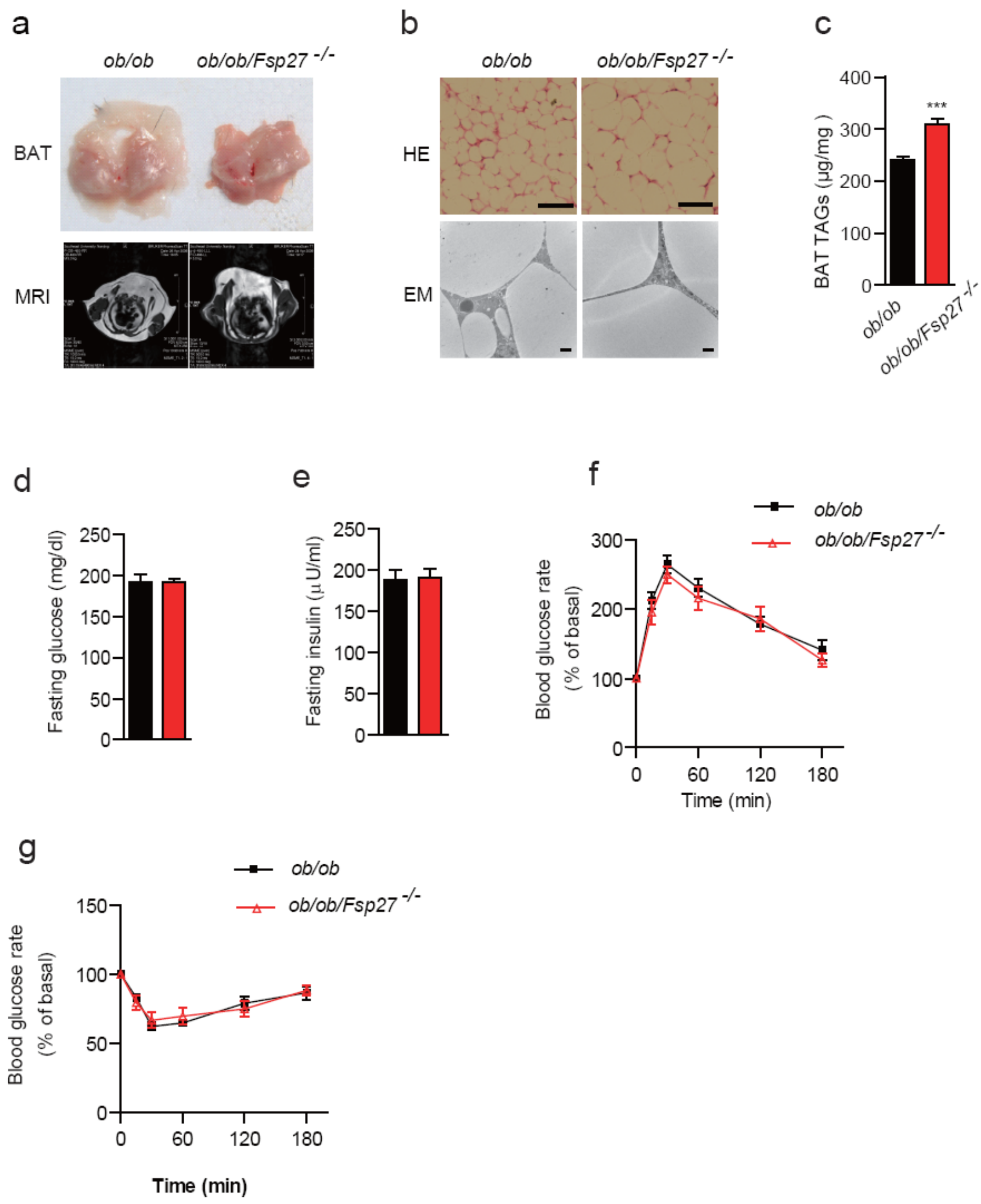

Supplementary Figure 3: Increased TAG storage in the BAT of *ob/ob/Fsp27*$^{-/-}$ mice. 4 month old chow fed *ob/ob* and *ob/ob/Fsp27*$^{-/-}$ mice were used. (a) Photographs (top panel) and MRI analysis of the BAT. (b) Morphology of BAT from *ob/ob* and *ob/ob/Fsp27*$^{-/-}$ mice. H&E, hematoxylin and eosin staining. EM, electron microscope image. Scale bar= 64 µm and 2µm for HE and EM respectively. (c) TAG concentration in the BAT of *ob/ob* and *ob/ob/Fsp27*$^{-/-}$ mice (n=5). (d) Fasting glucose and (e) fasting insulin concentrations of chow fed 4 month-old *ob/ob* (n=6) and *ob/ob/Fsp27*$^{-/-}$(n=6) mice. (f) Glucose tolerance tests (GTT) and (g) insulin tolerance tests (ITT) in chow fed 4 month-old *ob/ob* (n=5) and *ob/ob/Fsp27*$^{-/-}$ (n=5) mice.Quantitative data are presented as mean ± SEM. Significance was established using a 2-tailed Student’s t test. Differences were considered significant at P<0.05. *** indicates P<0.001.

Supplementary Figure 4

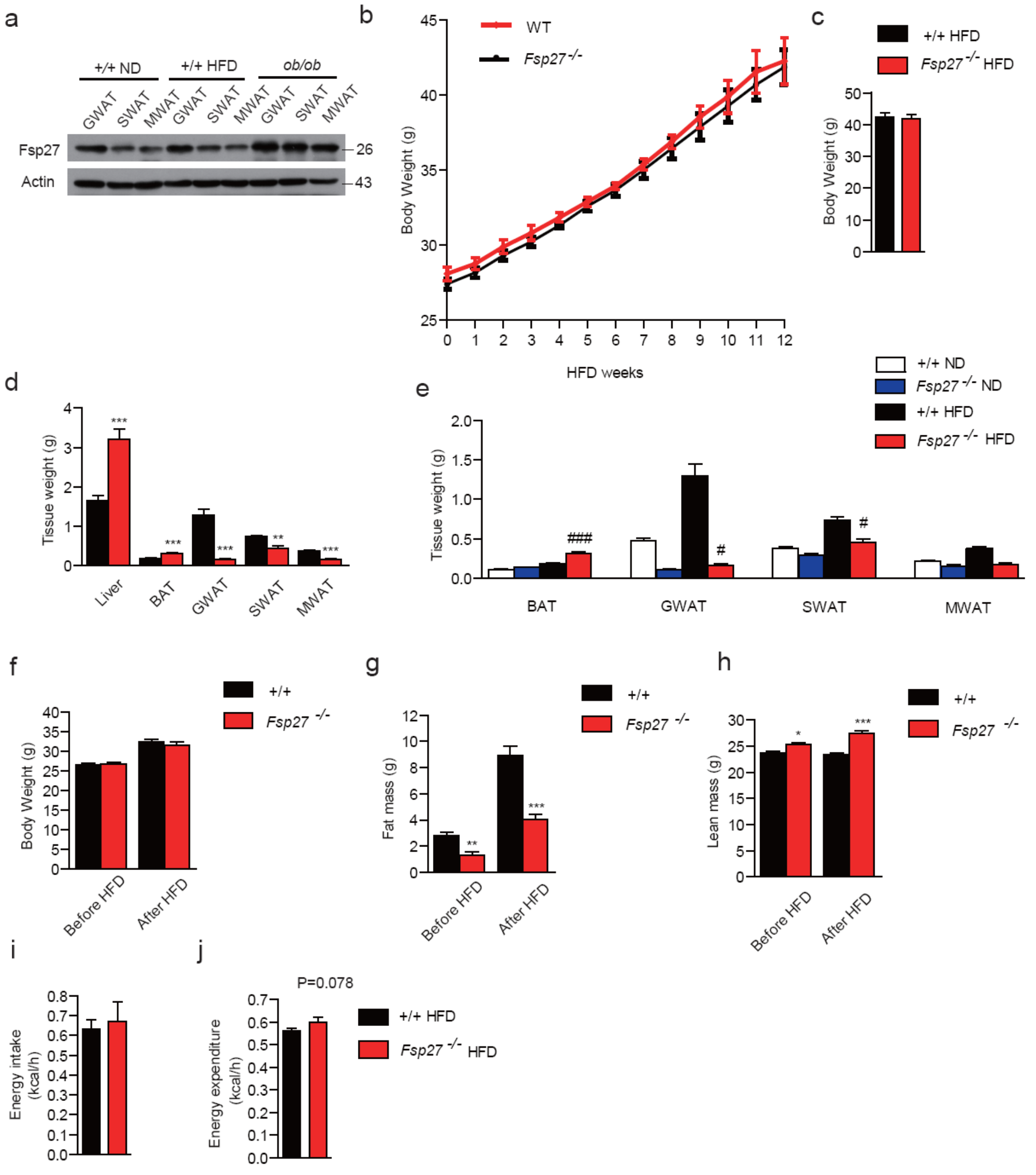

Supplementary Figure 4: Reduced fat mass in HFD fed *Fsp27*$^{-/-}$ mice. 3 month old WT and *Fsp27*$^{-/-}$mice were fed a chow diet (ND) or challenged with a HFD (D12331, 58% kcal of fat) for 3 months, n=4 per group (a-e). (a) Representative Fsp27 protein expression in gonadal fat (GWAT), subcutaneous fat (SWAT) and mesenteric fat (MWAT) from chow (ND), HFD fed or *ob/ob* mice. (b) Growth curves (body weight) of WT and *Fsp27*$^{-/-}$ mice fed with a HFD. (c) Body weight of WT and *Fsp27*$^{-/-}$ mice after 3 months on a HFD. (d) Tissue weights of WT and *Fsp27*$^{-/-}$ mice on a HFD. (e) Tissue weights of WT and *Fsp27*$^{-/-}$ mice fed a chow (ND) or HFD. 3 month old WT and *Fsp27*$^{-/-}$ mice were challenged with a HFD (D12492, 60% kcal of fat) for 6 weeks, n=6, (f-j). (f-h) Body weight, fat mass and lean mass of WT and *Fsp27*$^{-/-}$ mice on a HFD. (i) Energy intake (j) Energy expenditure of WT and *Fsp27*$^{-/-}$ mice on a HFD. Quantitative data are presented as mean ± SEM. Significance was established using a 2-tailed Student's t test. Differences were considered significant at $P<0.05$. *** indicates $P<0.001$. # indicates the difference between *Fsp27*$^{-/-}$ ND and *Fsp27*$^{-/-}$ HFD. # indicates $P<0.05$, ### indicates $P<0.001$.

Supplementary Figure 5

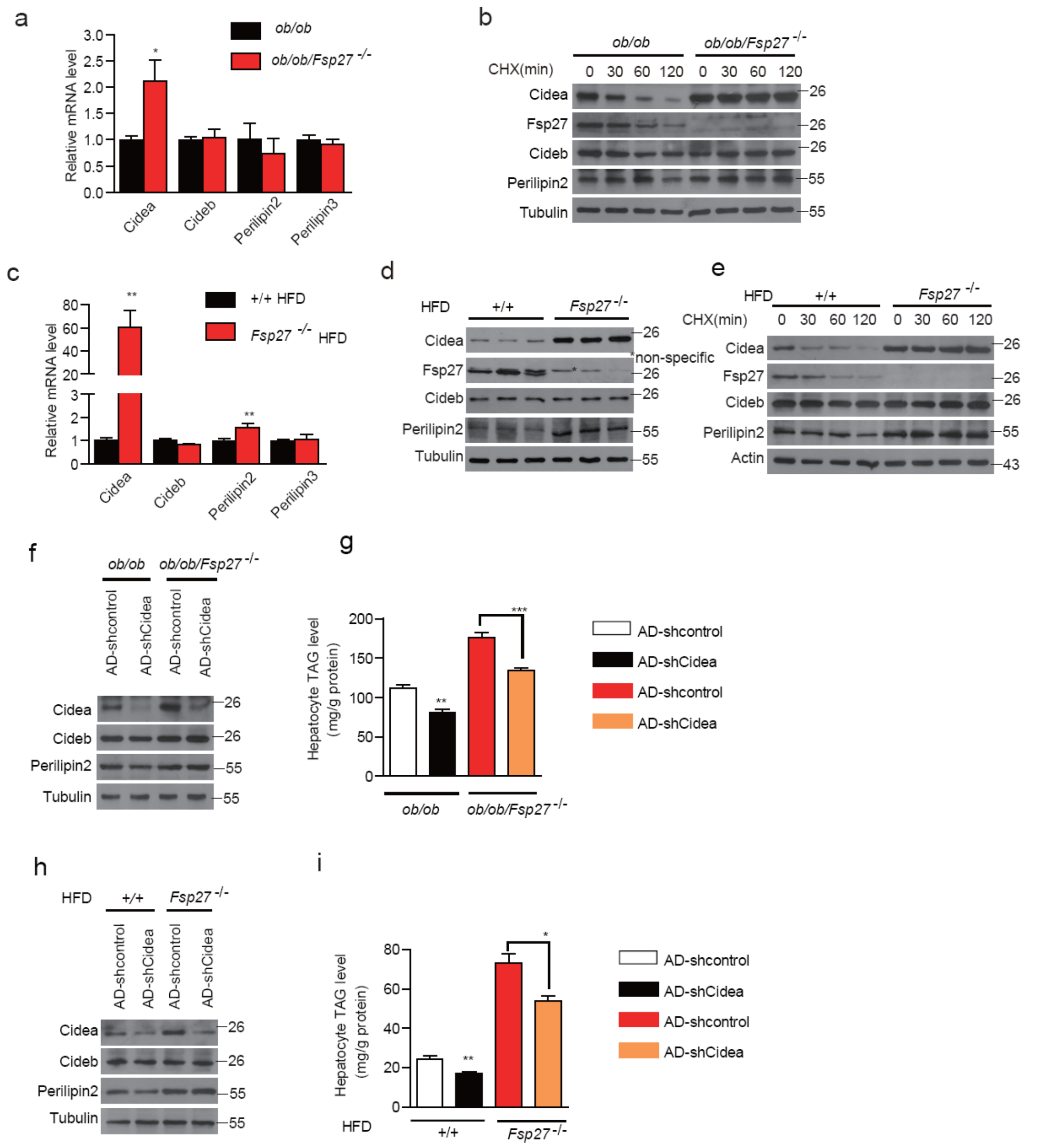

Supplementary Figure 5: Increased liver Cidea expression in *Fsp27*$^{-/-}$ mice on an *ob/ob* background or when fed a HFD. (a) Relative mRNA expression in the livers of 4 month old chow fed *ob/ob* and *ob/ob/Fsp27*$^{-/-}$ mice. (n=4). (b) Protein stability in isolated hepatocytes from 4 month old chow fed *ob/ob* and *ob/ob/Fsp27*$^{-/-}$ mice. 3 month old WT and *Fsp27*$^{-/-}$ mice were challenged with a HFD (D12331) for 3 months (c-e, h,i). (c) Relative mRNA and (d) protein expression. (e) protein stability in the hepatocytes isolated from HFD fed WT and *Fsp27*$^{-/-}$ mice. (f) Protein expression and (g) TAG concentrations in isolated hepatocytes from 4 month old *ob/ob* and *ob/ob/Fsp27*$^{-/-}$ mice infected with shCidea or shControl adenoviral vectors (n=4). (h) Protein expression and (i) TAG concentrations in isolated hepatocyte from HFD fed WT and *Fsp27*$^{-/-}$ mice treated with shCidea or shControl adenoviral vectors (n=4). Quantitative data are presented as mean ± SEM. Significance was established using a 2-tailed Student's t test. Differences were considered significant at $P<0.05$.* indicates $P<0.05$, ** indicates $P<0.01$, *** indicates $P<0.001$.

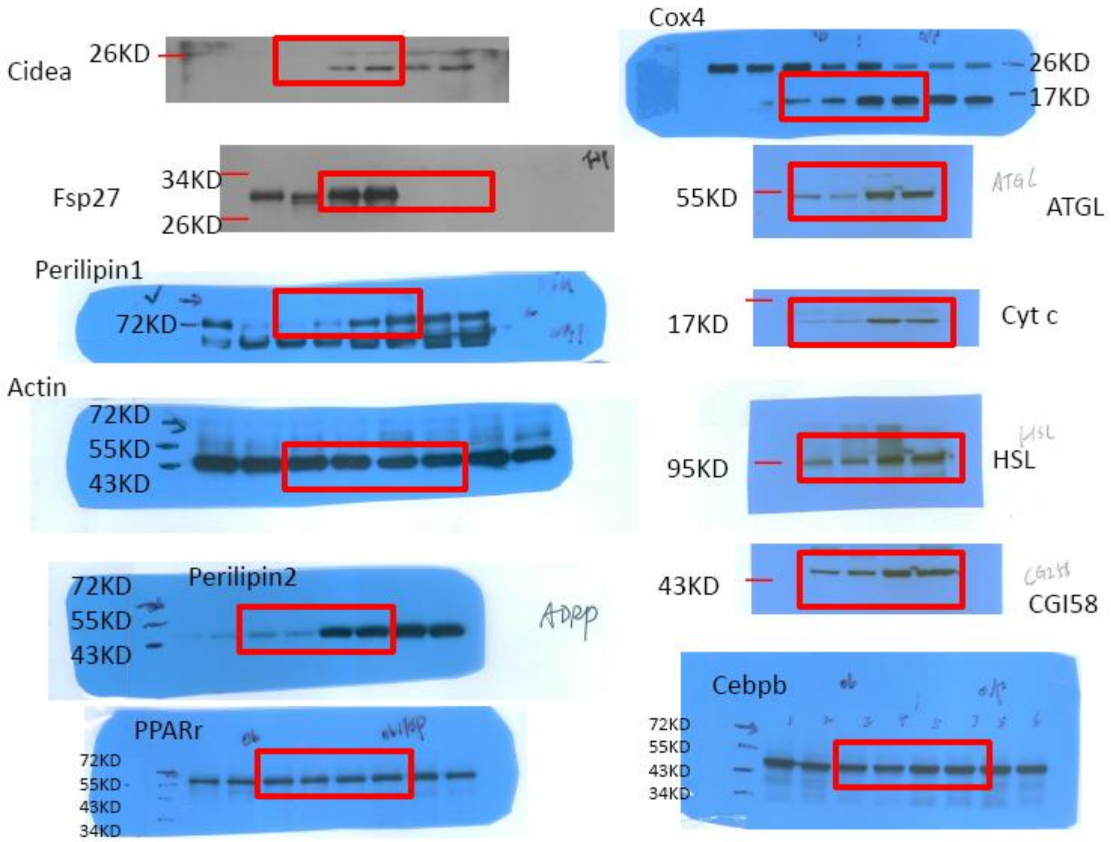

Supplementary Figure 6: Original western blots for images used in Figure1.

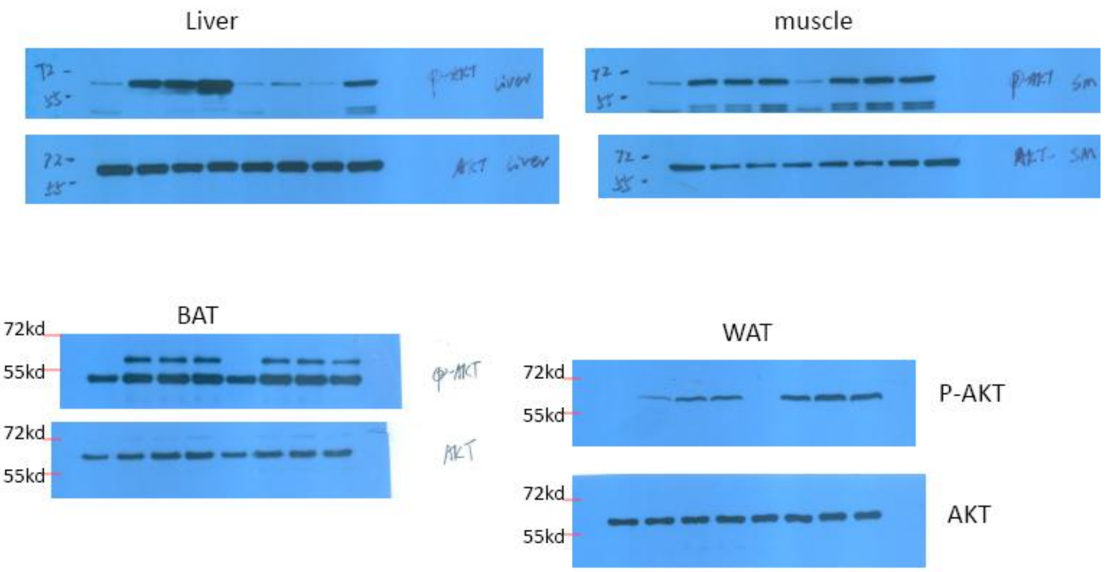

Supplementary Figure 7: Original western blots for images used in Figure4.

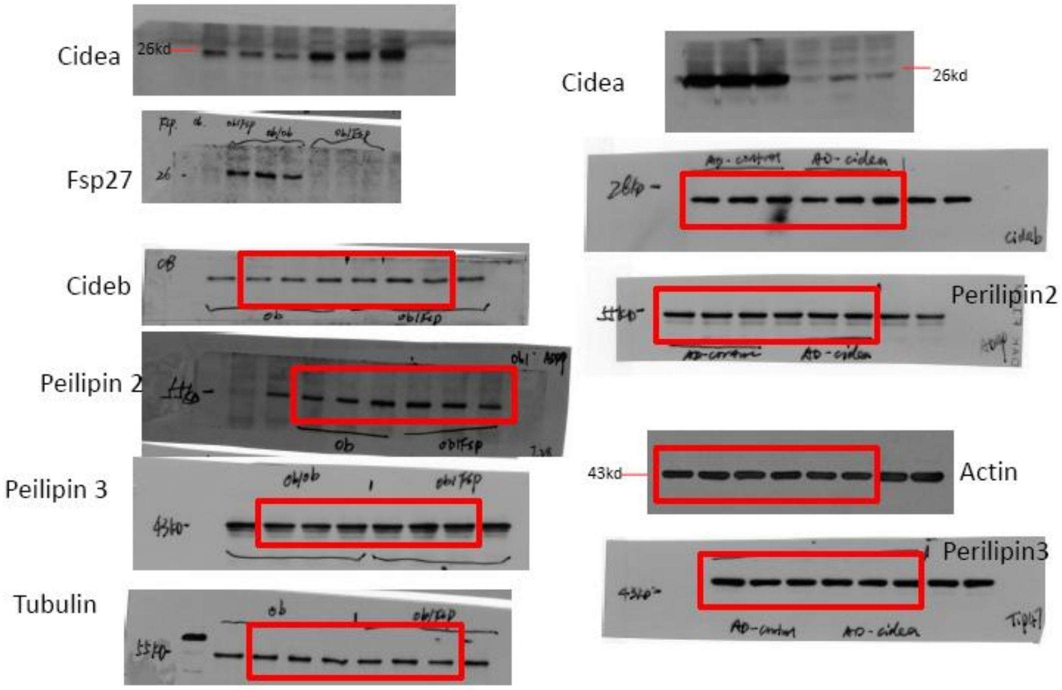

Supplementary Figure 8: Original western blots for images used in Figure7.

Supplementary Table1

ob/ob/Fsp27$^{-/-}$ vs. *ob/ob*

Up-Regulated Pathways: LFC > 0.5 && p.val < 0.05					
Pathway	positive	measured	total	%	Z Score
Electron Transport Chain	91	100	116	91.00%	21.41
Oxidative phosphorylation	52	59	65	88.14%	15.75
TCA Cycle	27	31	45	87.10%	11.22
Fatty Acid Beta Oxidation	26	34	46	76.47%	10.02
Fatty Acid Biosynthesis	16	22	26	72.73%	7.56
Mitochondrial LC-Fatty Acid Beta-Oxidation	13	16	21	81.25%	7.39
Amino Acid metabolism	37	92	205	40.22%	6.78
Glycolysis and Gluconeogenesis	23	48	70	47.92%	6.37
Triacylglyceride Synthesis	13	23	26	56.52%	5.55
Adipogenesis	42	132	134	31.82%	5.43
Heme Biosynthesis	6	9	21	66.67%	4.32
Glycogen Metabolism	14	34	42	41.18%	4.26
Arachidonate Epoxygenase Epoxide Hydrolase	3	3	13	100.00%	4.1
Nuclear Receptors	12	38	38	31.58%	2.84
Cholesterol Biosynthesis	6	15	30	40.00%	2.69
Tryptophan metabolism	12	43	48	27.91%	2.35
Diurnally regulated genes with circadian orthologs	13	48	48	27.08%	2.32
One carbon metabolism and related pathways	12	45	86	26.67%	2.17
Kennedy pathway	5	14	29	35.71%	2.15
Oxidative Stress	7	23	29	30.43%	2.05
Pentose Phosphate Pathway	3	7	19	42.86%	2.05
Acetylcholine Synthesis	3	7	17	42.86%	2.05
Mitochondrial Gene Expression	6	19	23	31.58%	2.01

Down-Regulated Pathways: LFC > 0.5 && p.val < 0.05					
Pathway	positive	measured	total	%	Z Score
B Cell Receptor Signaling Pathway	61	155	157	39.35%	7.63
Chemokine signaling pathway	63	180	198	35.00%	6.65
IL-5 Signaling Pathway	30	69	70	43.48%	5.97
IL-3 Signaling Pathway	37	99	101	37.37%	5.53
Kit Receptor Signaling Pathway	28	67	67	41.79%	5.51
T Cell Receptor Signaling Pathway	45	132	134	34.09%	5.38
Focal Adhesion	57	184	192	30.98%	5.24
Toll-like receptor signaling pathway	34	94	98	36.17%	5.07
DNA Replication	18	41	47	43.90%	4.66
Cell cycle	30	86	90	34.88%	4.52
EGFR1 Signaling Pathway	49	174	177	28.16%	4.08
G1 to S cell cycle control	22	61	64	36.07%	4.05
IL-4 signaling Pathway	21	61	63	34.43%	3.7
Apoptosis	26	83	84	31.33%	3.57

Toll Like Receptor signaling	13	33	33	39.39%	3.48
Complement and Coagulation Cascades	20	60	64	33.33%	3.44
G13 Signaling Pathway	14	38	38	36.84%	3.31
Regulation of Actin Cytoskeleton	40	150	157	26.67%	3.28
IL-7 Signaling Pathway	15	44	45	34.09%	3.08
Type II interferon signaling (IFNG)	12	33	36	36.36%	3.01
MAPK signaling pathway	40	157	162	25.48%	2.95
Inflammatory Response Pathway	11	30	32	36.67%	2.92
Complement Activation, Classical Pathway	7	16	18	43.75%	2.89
Prostaglandin Synthesis and Regulation	11	31	40	35.48%	2.79
Hypertrophy Model	8	20	21	40.00%	2.78
Apoptosis Modulation by HSP70	7	17	18	41.18%	2.69
TGF-beta Receptor Signaling Pathway	37	150	152	24.67%	2.61
Endochondral Ossification	18	62	67	29.03%	2.59
Matrix Metalloproteinases	10	29	30	34.48%	2.55
Small Ligand GPCRs	7	18	19	38.89%	2.51
Nucleotide Metabolism	7	19	36	36.84%	2.34
Senescence and Autophagy	25	98	100	25.51%	2.33
Glucuronidation	5	12	33	41.67%	2.3
IL-2 Signaling Pathway	20	76	76	26.32%	2.23
Integrin-mediated cell adhesion	24	97	102	24.74%	2.11
IL-1 Signaling Pathway	11	37	38	29.73%	2.11
Homologous recombination	5	13	13	38.46%	2.09
Androgen Receptor Signaling Pathway	26	109	114	23.85%	1.99
Signaling of Hepatocyte Growth Factor Receptor	10	34	34	29.41%	1.97

Supplementary Table1: The most significantly up-regulated and down-regulated pathways in the GWAT of *ob/ob/Fsp27*$^{-/-}$ mice were identified using Wiki pathway analysis. The 'total' column presents the total number of genes in that particular pathway. The 'measured' column provides the number of genes in a particular pathway whose expression differed (using the criteria described above), and the 'positive' column has the number of up-regulated or down-regulated genes. The Z score is the standard statistical test under a hypergeometric distribution. The pathways highlighted in green were significantly different in supplementary tables 1 and 2 whereas those in yellow were only different in supplementary table 1 or 2. LFC means log ratio of fold change.

Supplementary Table2

Fsp27$^{-/-}$ vs. WT

Up-Regulated Pathways: LFC > 0.5 && p.val < 0.05					
Pathway	positive	measured	total	%	Z Score
Electron Transport Chain	64	81	116	79.01%	19.7
Oxidative phosphorylation	37	48	65	77.08%	14.65
TCA Cycle	23	27	45	85.19%	12.29
Fatty Acid Beta Oxidation	22	30	46	73.33%	10.88
Mitochondrial LC-Fatty Acid Beta-Oxidation	12	15	21	80.00%	8.5
Amino Acid metabolism	29	71	205	40.85%	8.06
Glycolysis and Gluconeogenesis	19	36	70	52.78%	7.99
Fatty Acid Biosynthesis	12	20	26	60.00%	6.97
Arachidonate Epoxygenase Epoxide Hydrolase	3	3	13	100.00%	4.89
Tryptophan metabolism	10	28	48	35.71%	4.15
Synthesis and Degradation of Ketone Bodies	2	4	8	50.00%	2.47
Eicosanoid Synthesis	4	14	36	28.57%	2.08
Nuclear receptors in lipid metabolism and toxicity	4	15	40	26.67%	1.92
Cholesterol Biosynthesis	4	15	30	26.67%	1.92

Down-Regulated Pathways: LFC > 0.5 && p.val < 0.05					
Pathway	positive	measured	total	%	Z Score
Cytoplasmic Ribosomal Proteins	45	76	82	59.21%	9.56
Complement Activation, Classical Pathway	10	10	18	100.00%	6.8
Complement and Coagulation Cascades	16	28	64	57.14%	5.46
Focal Adhesion	45	128	192	35.16%	5.25
Estrogen metabolism	7	10	29	70.00%	4.32
Inflammatory Response Pathway	9	16	32	56.25%	4.03
Glucuronidation	7	11	33	63.64%	3.98
Endochondral Ossification	16	41	67	39.02%	3.57
Senescence and Autophagy	25	76	100	32.89%	3.48
Aflatoxin B1 metabolism	3	4	11	75.00%	2.99
Myometrial Relaxation and Contraction Pathways	29	102	161	28.43%	2.85
Dopminergic Neurogenesis	4	7	32	57.14%	2.72
Striated Muscle Contraction	7	16	45	43.75%	2.72
Prostaglandin Synthesis and Regulation	9	23	40	39.13%	2.68
Hypertrophy Model	6	13	21	46.15%	2.67
Regulation of Actin Cytoskeleton	25	93	157	26.88%	2.32
SHH, FGF8, Stat3	1	1	6	100.00%	2.15
Glucocorticoid & Mineralcorticoid Metabolism	1	1	31	100.00%	2.15
Irinotecan Pathway	4	9	13	44.44%	2.09
Ptf1a related regulatory pathway	3	6	14	50.00%	2.06
TGF Beta Signaling Pathway	11	36	52	30.56%	2.01
Alpha6-Beta4 Integrin Signaling Pathway	14	49	67	28.57%	1.98

Supplementary Table2: The most significantly up-regulated and down-regulated pathways in the GWAT of *Fsp27*$^{-/-}$ mice were identified using Wiki pathway analysis. Data is presented in the same manner as in Supplementary table 1.

Supplementary Table 3: Sequences of primers used for qRT-PCR analysis.

	Forward (5'-3')	Reverse(5'-3')
Actin	GGCTGTATTCCCCTCCATCG	CCAGTTGGTAACAATGCCATGT
ACC1	AGCTGATCCTGCGAACCT	GCCAAGCGGATGTAAACT
FAS	TCCAAGACTGACTCGGCTACTGAC	GCAGCCAGGTTCGGAATGCTATC
SCD1	TTCTTGCGATACACTCTGGTGC	CGGGATTGAATGTTCTTGTCGT
Elovl6	AAGCAGTTCAACGAGAACGAA	CGTACAGCGCAGAAAACAGG
DGAT1	TCCGTCCAGGGTGGTAGTG	TGAACAAAGAATCTTGCAGACGA
DGAT2	CTGGCTGATAGCTGCTCTCTACTTC	TGTGATCTCCTGCCACCTTTC
SREBP1c	GGAGCCATGGATTGCACATT	GCTTCCAGAGAGGAGGCCAG
SREBP2	CCCTTGACTTCCTTGCTGCA	GCGTGAGTGTGGGCGAATC
Pparg	TTGACAGGAAAGACAACGGAC	CTTCTACGGATCGAAACTGG
Ppara	ACGGCAATGGCTTTATCA	CGCTGCGTCGGACTCGGT
LXRα	ATGTCCACGAGTGACTGTT	TTGACTCTCCCTTAATGCTAC
LXRβ	TATGCCTTGCTTATCGCCATC	CTTGTCCTGGAGTCGCAATG
CPT1	ACCACTGGCCGCATGT	CTCCATGGCGTAGTAGTTGCT
CPT2	CAGCACAGCATCGTACCCA	TCCCAATGCCGTTCTCAAAAT
Cox4	CGGCGTGACTACCCCTTG	TGAGGGATGGGGCCATACA
Cyto C	CCAAATCTCCACGGTCTGTTC	ATCAGGGTATCCTCTCCCCAG
Acadl	GCATCAACATCGCAGAGAAA	ACGCTTGCTCTTCCCAAGTA
Acadm	GCTAGTGGAGCACCAAGGAG	CCAGGCTGCTCTCTGGTAAC
Pck1	GAAGGACAAAGATGGCAAGTT	CGTTTTCCTTAGGGATGTAGC
G6pc	CTGAGCGCGGGCATCATAAT	GATTCTTAGGATCGCCCAGAAAG
CRP	ATGGAGAAGCTACTCTGGTGC	ACACACAGTAAAGGTGTTCAGTG
IL-1β	GCAACTGTTCCTGAACTCAACT	ATCTTTTGGGGTCCGTCAACT
CD11c	ACACAGTGTGCTCCAGTATGA	GCCCAGGGATATGTTCACAGC
F4/80	CTTTGGCTATGGGCTTCCAGTC	GCAAGGAGGACAGAGTTTATCGTG
MCP1	AGGTCCCTGTCATGCTTCTG	GCTGCTGGTGATCCTCTTGT
MIP1α	TTCTCTGTACCATGACACTCTGC	CGTGGAATCTTCCGGCTGTAG
TNFα	CCAGACCCTCACACTCAGATC	CACTTGGTGGTTTGCTACGAC
SAA3	CCTGGGCTGCTAAAGTCATC	ACCCAGTAGTTGCCCCTCTT
IL-4	ATGGAGCTGCAGAGACTCTT	AAAGCATGGTGGCTCAGTAC

IL-6	CCAGAGATACAAAGAAATGATGG	ACTCCAGAAGACCAGAGGAAAT
IL-10	TGAATTCCCTGGGTGAGAAG	TCACTCTTCACCTGCTCCACT
IL-18	GACTCTTGCGTCAACTTCAAGG	CAGGCTGTCTTTTGTCAACGA
Arg1	ATGGAAGAGACCTTCAGCTAC	GCTGTCTTCCCAAGAGTTGGG
Ym1	GGGCATACCTTTATCCTGAG	CCACTGAAGTCATCCATGTC
Adiponectin	TGTTCCTCTTAATCCTGCCCA	CCAACCTGCACAAGTTCCCTT
Cidea	TCCTCGGCTGTCTCAATG	TGGCTGCTCTTCTGTATCG
Cideb	TCTGTGATCATAAGCGGACA	GCAGCAGCGAGGAAGTCCAA
Perilipin2	GATTGAATTCGCCAGGAAGA	TGGCATGTAGTCTGGAGCTG
Perilipin3	CTGAGAAAGGCGTCAAGACC	TTTCTTGAGCCCCAGACACT
ASC	CTTGTCAGGGGATGAACTCAAAA	GCCATACGACTCCAGATAGTAGC
NLRP3	ATTACCCGCCCGAGAAAGG	TCGCAGCAAAGATCCACACAG
Caspase-1	ACAAGGCACGGGACCTATG	TCCCAGTCAGTCCTGGAAATG
TXNIP	TCTTTTGAGGTGGTCTTCAACG	GCTTTGACTCGGGTAACTTCACA
